# Anlotinib plus sintilimab as first-line treatment for patients with advanced colorectal cancer (APICAL-CRC): an open-label, single-arm, phase II trial

**DOI:** 10.1038/s41392-025-02383-9

**Published:** 2025-09-16

**Authors:** Zhan Wang, Bao-Dong Qin, Chen-Yang Ye, Miao-Miao Wang, Ling-Yan Yuan, Hou-Shan Yao, Xiao-Dong Jiao, Ke Liu, Wen-Li Zhou, Wen-Xing Qin, Li Sun, Wei-Ping Dai, Yan Ling, Ying Wu, Shi-Qi Chen, Ying-Fu Zhang, Dong-Min Shi, Xiao-Peng Duan, Xue Zhong, Xi He, Wen-Xin Zhai, Bei Zhang, Da-Dong Zhang, Ning Gao, Yuan-Sheng Zang

**Affiliations:** 1 Department of Medical Oncology, Changzheng Hospital, Naval Medical University, Shanghai, China; 2 Department of General Surgery, Changzheng Hospital, Naval Medical University, Shanghai, China; 3 D Medicines Inc., Shanghai, China

**Keywords:** Drug development, Gastrointestinal cancer

## Abstract

This is an investigator-initiated, open-label, single-arm, phase II trial that aimed to assess the combination of sintilimab plus anlotinib among patients with treatment-naïve metastatic colorectal cancer (mCRC) (APICAL-CRC ClinicalTrials.gov number, NCT04271813). Between June 2020 and September 2023, a total of 30 patients were eventually enrolled and received the study regimen. Among these 30 patients, 50% had an Eastern Cooperative Oncology Group(ECOG) score of 0–1, and the other 50% had a score of 2. The objective response rates (ORRs) were 48.3% (95% CI 29.4–67.5) in the efficacy-evaluable cohort and 46.7% (95% CI 28.3–65.7) in the intent-to-treat (ITT) cohort. Twelve patients had stable disease, and the disease control rates (DCRs) were 89.7% (95% CI 72.6–97.8) and 86.7% (95% CI 69.3–96.2) in the efficacy-evaluable and ITT cohorts, respectively. The median progression-free survival (mPFS) was 8.6 months (95% CI 4.8–11.0), and the median overall survival (mOS) reached 22.9 months (95% CI 13.5–36.3). Treatment-related adverse events (TRAEs) of any grade were reported in 23 patients (76.7%), and grade 3 TRAEs occurred in 4 patients (13.3%). Multivariate Cox regression analysis revealed that the presence of liver metastases was an independent prognostic factor for poor PFS (HR = 5.66, 95% CI 1.58–20.2) and OS (HR = 7.85, 95% CI 1.38–44.8), whereas *FLT* mutation was independently associated with poor OS(HR = 12.5, 95% CI 1.54–101). This trial demonstrated that sintilimab plus anlotinib exhibited promising antitumor efficacy along with a manageable safety profile among treatment-naïve mCRC patients.

## Introduction

Colorectal cancer (CRC) is the third most common malignancy and the second leading cause of cancer-related death worldwide.^1^ Despite recent advances in CRC research, approximately 15–30% of patients present with metastatic disease at the time of initial diagnosis, whereas an additional 20–50% of patients with initially localized CRC will eventually develop metastases.^2^ First-line metastatic CRC (mCRC) typical regimens were fluorouracil-based chemotherapy with anti-EGFR/VEGF biologics, but the prognosis of treated patients remains poor, with a median overall survival of approximately 2 years.^3,4^ Furthermore, certain mCRC patients may not qualify for standard doublet or triplet first-line chemotherapy in conjunction with targeted therapy because of various factors, such as advanced age, poor performance status, comorbidities, or personal preference.^5^ Exploring novel, highly effective, and low-toxicity therapeutic regimens holds critical clinical importance for these patients.

In the past few years, immune checkpoint inhibitors have emerged as promising treatments for mCRC, particularly for ~5% of patients harboring DNA mismatch repair deficiencies or microsatellite instability-high (dMMR/MSI-H) status.^6–8^ However, a majority of pMMR/MSS mCRC patients are intrinsically resistant to immune checkpoint inhibitors due to the immune desert or immune-excluded microenvironment.^9^ To increase the efficacy of immune checkpoint inhibitors in this context, a compelling research approach is to combine antitumor agents with immunomodulatory properties aimed at improving the immunogenicity of these tumors.^10^ Antiangiogenic agents have the ability to reprogram immunosuppressive microenvironments into immunostimulatory microenvironments, either directly or indirectly, suggesting that the combination of immune checkpoint inhibitors with antiangiogenic agents could yield synergistic antitumor effects.^11,12^ For example, the REGONIVO trial revealed that combining regorafenib with nivolumab resulted in a manageable safety profile and encouraging antitumor activity in refractory MSS colorectal cancer.^13^ However, the LEAP-017 trial focused on heavily pretreated, chemotherapy-refractory mCRC patients comparing lenvatinib plus pembrolizumab with regorafenib or TAS-102, which failed to meet its prespecified primary endpoint.^14^ Patient characteristics (e.g., prior therapy), treatment patterns (e.g., subsequent therapies), and the performance of the control arm may affect the results of the LEAP-017 trial. While the clinical utility of this regimen in later-line settings remains uncertain, the potential for this strategy to provide clinical benefit in treatment-naïve patients with intact immune function warrants exploration.

Anlotinib, an oral tyrosine kinase inhibitor (TKI) that targets VEGFR1/2/3, FGFR1/2/3, PDGFR, and c-Kit, has a broad spectrum of effective antitumor activities against various types of cancers.^15–17^ Several trials have identified anlotinib as a potential candidate for mCRC patients.^18,19^ A retrospective study assessed the feasibility and tolerability of anlotinib plus a PD-1 inhibitor for patients with chemorefractory mCRC, and the results demonstrated encouraging efficacy and an acceptable safety profile in mCRC patients.^20^ Sintilimab, a fully human IgG4 monoclonal antibody that inhibits the interaction between PD-1 and its ligands, has demonstrated clinical benefit in a range of cancers, including non-small cell lung cancer and hepatocellular carcinoma.^21,22^ Previous trials have underscored the potential of sintilimab in combination with anlotinib as a novel chemotherapy-free regimen for conditions such as cervical cancer and lung cancer.^23,24^ In addition, the combination of sintilimab with fruquintinib has shown promise in refractory CRC patients, further suggesting the potential benefit of combining sintilimab with antiangiogenic TKIs.^25^

On the basis of the above findings, the combination of immune checkpoint inhibitors with antiangiogenic TKIs is expected to yield strong synergistic antitumor effects as a first-line regimen for mCRC. This approach could pave the way for a “chemotherapy-free” regimen in the first-line treatment of mCRC in the presence of a healthy immune system. Therefore, this study was designed to investigate the efficacy and safety of first-line sintilimab plus anlotinib in mCRC patients.

## Results

### Patients

**Fig. 1:**
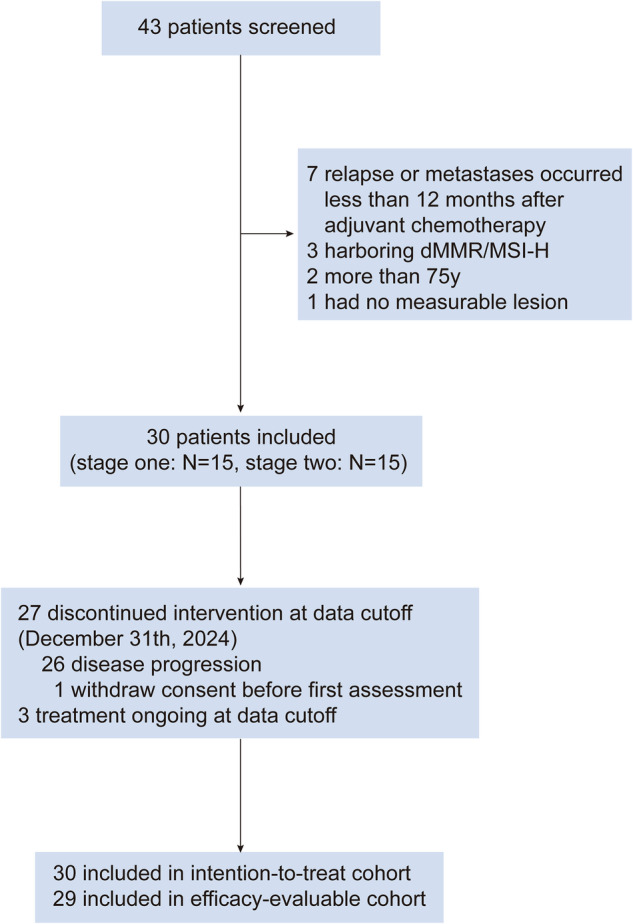
APICAL-CRC study design

Between June 2020 and September 2023, a total of 43 patients were screened for eligibility; 30 patients eventually enrolled and received the study regimen, constituting the intent-to-treat (ITT) population and safety population (Fig. 1). Of the 15 patients with treatment-naïve mCRC enrolled in the first stage of this trial, 8 achieved an objective response. Subsequently, 15 additional patients were included in the second stage. All patients were administered sintilimab plus anlotinib as a first-line treatment. Among these patients, 15 out of 30 (50.0%) had an Eastern Cooperative Oncology Group (ECOG) score of 0–1. The baseline characteristics of patients with ECOG scores 0–1 and 2 were further described (Supplementary Table 1). Thirteen patients (43.3%) had more than 2 metastatic sites. Among the 9 (30%) patients with lung metastases, 3 had isolated lung metastases. Among the 19 (63.3%) patients with liver metastases, 11 had isolated liver metastases. All patients were MSS/pMMR, 15 harbored KRAS mutations, and 1 harbored BRAF V600E mutation (Table 1).

**Table 1:** Patient characteristics at baseline

Characteristics	Patients (*N* = 30)
Age	
Median (Range)	60.5 (38–75)
Sex	
Female	13 (43.3%)
Male	17 (56.7%)
ECOG performance status	
0	1 (3.3%)
1	14 (46.7%)
2	15 (50.0%)
Primary site	
Left Colon	6 (20.0%)
Right Colon	10 (33.3%)
Rectum	14 (46.7%)
Number of metastatic sites (Range)	
1	17 (56.7%)
≥2	13 (43.3%)
Metastatic site	
Liver	19 (63.3%)
Lung	9 (30.0%)
Distant lymph node	11 (36.7%)
Peritoneum	5 (16.7%)
Other	7 (23.3%)
Isolated lung metastases	3 (10%)
Number of lesions, median (Range)	6 (4 to 8)
Isolated liver metastases	11 (36.7%)
Number of lesions, median (Range)	4 (1–50)
MMR status	
dMMR	0 (0%)
pMMR	30 (100%)
KRAS mutation type	
Yes	15 (50.0%)
No	15 (50.0%)
BRAF V600E mutation	
Yes	1 (3.3%)
No	29 (96.7%)
PD-L1 expression	
TPS < 1	25 (83.3%)
TPS ≥ 1	2 (6.7%)
Unknown	3 (10.0%)
TMB (Mutation/Mb), median (Range)	8.38 (2.79–16.8)

One patient discontinued treatment before the first radiological assessment due to withdrawal, while the remaining 29 patients received at least one scheduled postbaseline assessment after treatment with sintilimab plus anlotinib as the efficacy-evaluable cohort. The data cutoff for efficacy and safety analyses was December 31, 2024, with a median follow-up of 37.2 months (95% CI: 21.7–48.7). At the data cutoff, 3 patients were still receiving treatment, and 27 patients discontinued protocol treatment due to PD (*n* = 26) or withdrawal (*n* = 1). Among the 26 patients with confirmed disease progression, 25 received second-line therapies, including FOLFOX/FOLFIRI-based regimens combined with bevacizumab or cetuximab, capecitabine plus bevacizumab, and intensive regimens such as FOLFOXIRI [FOLFIRI combined with bevacizumab (*N* = 6), FOLFOX combined with bevacizumab (*N* = 5), FOLFIRI plus cetuximab (*N* = 6), capecitabine plus bevacizumab (*N* = 2), FOLFOXIRI plus bevacizumab (*N* = 1), XELOX + cetuximab (*N* = 1)]. Two patients underwent conversion surgery, and another 5 patients underwent radical treatment, including radiotherapy and ablation.

### Efficacy

**Fig. 2:**
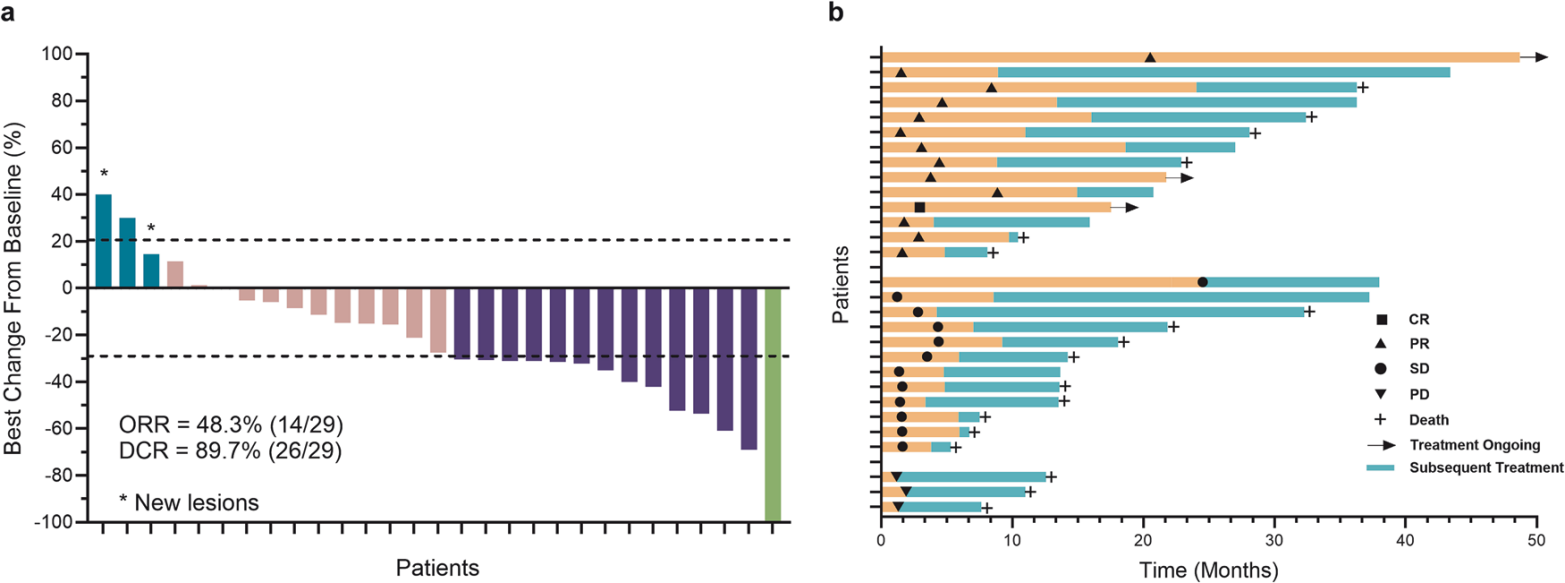
Clinical activity endpoints associated with the combination of sintilimab plus anlotinib as a first-line regimen in mCRC patients. **a** The best percentage change from baseline with respect to the tumor burden (defined as the sum of the diameters of all target lesions) from 29 mCRC patients in the efficacy-evaluable cohort. The upper dashed line indicates a 20% increase in tumor burden (PD), and the lower dashed line indicates a 30% decrease in tumor burden (PR). Asterisks indicate the presence of a new lesion. **b** Duration of drug exposure in the efficacy-evaluable population. The length of each bar represents the treatment duration for each patient

Among the 29 patients eligible for efficacy analyses, 1 achieved a complete response, and 13 exhibited confirmed partial responses. The objective response rates (ORRs) were 48.3% (95% CI 29.4–67.5) in the efficacy-evaluable cohort and 46.7% (95% CI 28.3–65.7) in the ITT cohort (Fig. 2a). The median time to response was 2.8 months (range: 1.4–20.5). The median time to best response was 4.6 months (95% CI 2.8–8.4). Among the 14 patients with objective responses, responses were discontinued in 11 patients at the data cutoff, with a median response duration of 8.1 months (range: 2.2–15.6) (Fig. 2b). Twelve patients had stable disease, and the disease control rates (DCRs) were 89.7% (95% CI 72.6–97.8) and 86.7% (95% CI 69.3–96.2) in the efficacy-evaluable and ITT cohorts, respectively. Target tumor shrinkage per the RECIST criteria v1.1 was observed in 79.3% (23/29) of patients after baseline tumor assessment.

**Fig. 3:**
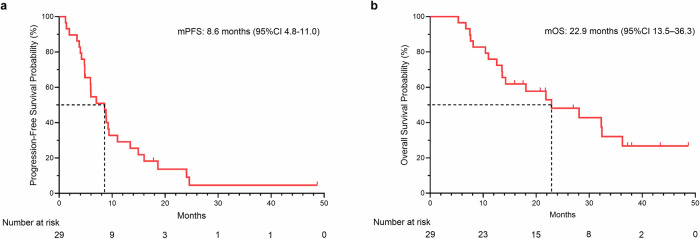
PFS Kaplan–Meier curve (**a**) and OS Kaplan–Meier curve (**b**). The data cutoff date for clinical activity was December 31, 2024, and the median follow-up interval was 37.2 months

Progression-free survival (PFS) events were recorded for 26 of 29 patients (89.7%) because of disease progression. The median PFS was 8.6 months (95% CI 4.8–11.0). The 6- and 12-month PFS rates were 54.6% and 29.1%, respectively (Fig. 3a). Overall survival (OS) events were recorded for 17 patients (58.6%). The median OS was 22.9 months (95% CI 13.5–36.3), and the 12- and 24-month OS rates were 75.9% and 48.1%, respectively (Fig. 3b).

Subgroup analyses revealed a positive association between efficacy and ECOG score and liver metastases. Patients with an ECOG score of 0–1 presented a much greater ORR (66.7% vs. 21.4%) and longer mPFS (11.0 months vs. 5.9 months) and mOS (36.3 months vs. 18.1 months) than patients with an ECOG score of 2 (Supplementary Fig. 1). Similarly, patients without liver metastases had an ORR of 70.0%, with a median PFS and OS of 14.9 months (95% CI 3.7–24.6), which were much better than those of patients with liver metastases (ORR = 36.8%; mPFS: 5.9 months, 95% CI 4.0–18.6). The mOS of patients without liver metastases has not yet been reached; that of those with liver metastases was 18.1 months (95% CI 10.4–28.1) (Supplementary Fig. 1).

### Safety

In the safety analysis cohort, 23 out of 30 patients experienced at least 1 treatment-related adverse event (TRAE) (Table 2). Nine patients developed a single TRAE, and 14 patients experienced two or more TRAEs during the therapeutic course. Common TRAEs included diarrhea, hypothyroidism, hypertension, and hand‒foot syndrome (HFS). Grade 3 TRAEs occurred in 4 patients. One patient developed grade 3 HFS and anemia, 1 patient developed grade 3 HFS, 1 patient experienced thrombocytopenia, and 1 patient developed immune-related pancreatitis. No grade-4 or -5 TRAEs or treatment-related deaths were observed. Three patients had anlotinib dose reduction due to grade 3 TRAEs. Most TRAEs were reversible either by reducing the dose of anlotinib or by administering appropriate medications. In addition, one patient temporarily interrupted sintilimab due to grade 3 immune-related pancreatitis but was subsequently rechallenged with sintilimab after recovering.

**Table 2:** Treatment-related adverse events

Adverse Event	*N*	Grade 1	Grade 2	Grade 3	Grade 4
Diarrhea	13 (43.3%)	10 (33.3%)	3 (10.0%)		
Hypothyroidism	9 (30.0%)	2 (6.7%)	7 (23.3%)		
Hypertension	7 (23.3%)	1 (3.3%)	6 (20.7%)		
Hand–Foot Syndrome	6 (20.0%)	4 (13.3%)		2 (6.9%)	
Anorexia	6 (20.0%)	6 (20.0%)			
Malaise	4 (13.3%)	4 (13.3%)			
Laryngitis	4 (13.3%)	3 (10.0%)	1 (3.3%)		
Constipation	3 (10.0%)	3 (10.0%)			
Rash	4 (13.3%)	4 (13.3%)			
Abdominal distension	3 (10.0%)	3 (10.0%)			
Granulocytopenia	3 (10.0%)	3 (10.0%)			
Pituitary insufficiency	2 (6.7%)		2 (6.7%)		
Insomnia	2 (6.7%)	2 (6.7%)			
Gum infection	2 (6.7%)	2 (6.7%)			
Fever	2 (6.7%)	2 (6.7%)			
Anemia	1 (3.3%)			1 (3.3%)	
Urinary tract infections	1 (3.3%)		1 (3.3%)		
Thrombocytopenia	1 (3.3%)			1 (3.3%)	
Pancreatitis	1 (3.3%)			1 (3.3%)	
ALT elevation	1 (3.3%)		1 (3.3%)		
Hyperthyroidism	1 (3.3%)		1 (3.3%)		
Nausea	1 (3.3%)		1 (3.3%)		
Asthenia	1 (3.3%)		1 (3.3%)		
Pneumonia	1 (3.3%)				
Weight gain	1 (3.3%)	1 (3.3%)			
Hyperamylasemia	1 (3.3%)	1 (3.3%)			
Weight loss	1 (3.3%)	1 (3.3%)			

### Exploratory analysis

**Fig. 4:**
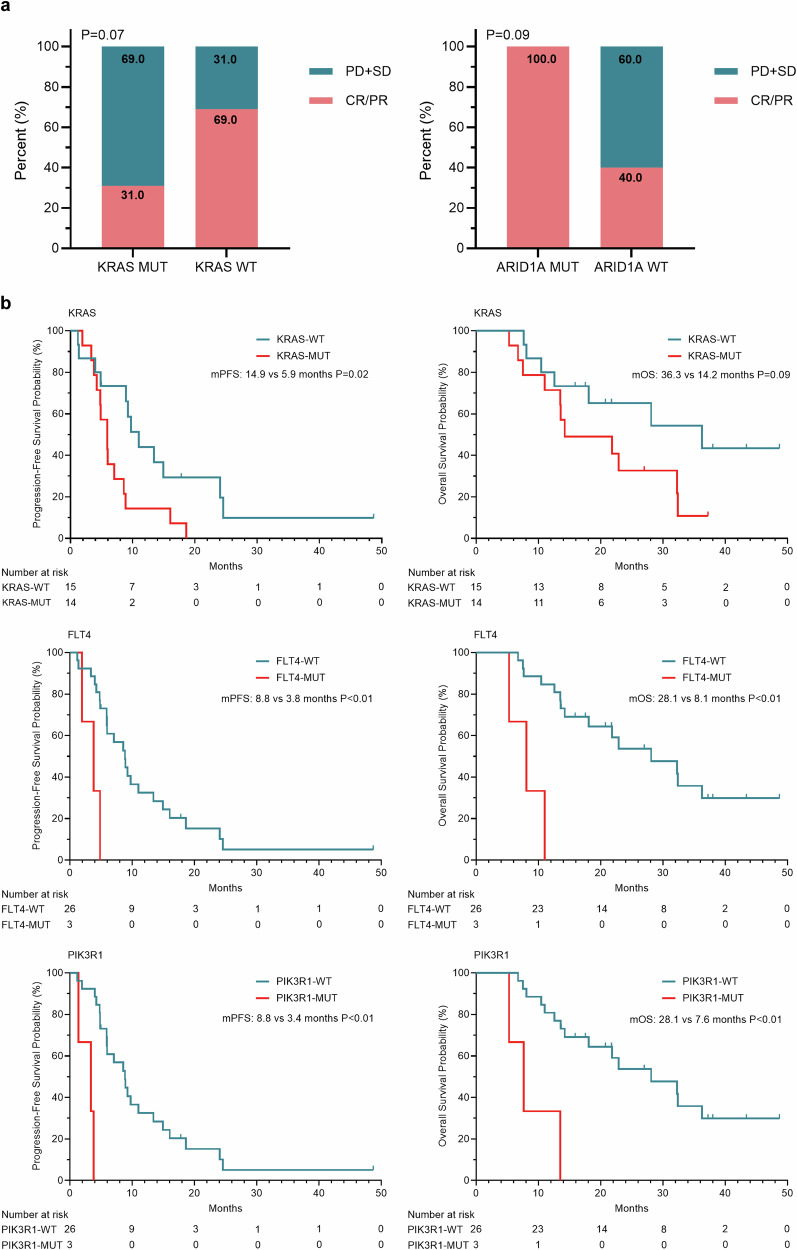
Prespecified exploratory analysis. **a** Association of KRAS and ARID1A mutations with the ORR. **b** Association between KRAS/FLT4/PIK3R1 mutations and clinical benefit from sintilimab plus anlotinib. *P*-value, Fisher’s exact test

**Fig. 5:**
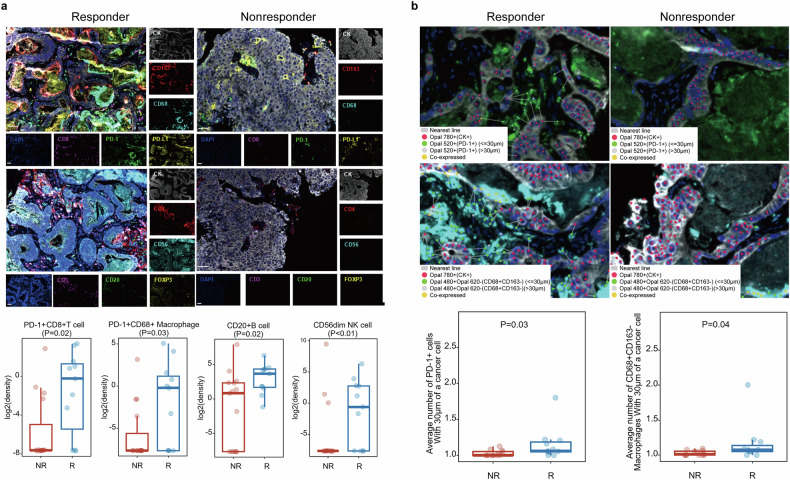
Prespecified exploratory analysis. **a** Correlations between the tumor immune microenvironment and response to the study regimen. Comparisons of the density of CD8+ PD-1+ T cells, PD-L1+ CD68+ macrophages, CD20+ B cells, and CD56dim NK cells between nonresponders and responders (nonresponders, PD + SD; responders, PR + CR). **b** Association between immune cell (PD-1+ cell, CD68+CD163− macrophage) infiltration within 30 μm of a cancer cell and the clinical response. *P* value, Wilcoxon test

Gene sequencing revealed that *TP53* was the most frequently altered gene, occurring in 85% (23/27) of patients, followed by *APC* (67%), *KRAS* (56%), *PTK6* (26%), and *SMAD4* (26%) (Supplementary Fig. 2). Enrichment analysis revealed that patients with *ARID1A* mutations had a relatively greater ORR than did wild-type (WT) patients (100% vs. 40%, *P* = 0.09), whereas those with *KRAS* mutations had a relatively lower ORR than did WT patients (31% vs. 69%, *P* = 0.07) (Fig. 4a). Patients harboring *KRAS*, *PIK3R1*, or *FLT4* mutations had significantly shorter PFS and OS than their WT counterparts did (*P* < 0.05 for all, Fig. 4b). Multiplex immunofluorescence further confirmed that PD-1+CD8+ T cells, PD-L1 +CD68+ macrophages, CD20+ B cells, and CD56dim NK cells were significantly enriched in the TME of responders (Fig. 5a). Moreover, a greater number of immune cells (PD-1+ cells and CD68+CD163^_^ macrophages) within 30 μm of cancer cells were observed among responders than among nonresponders (Fig. 5b), whereas no significant difference in tertiary lymphoid structure (TLS) was found (Supplementary Fig. 3). Baseline plasma exosomal miRNA analysis revealed that patients who achieved CR/PR had significantly lower levels of circulating small extracellular vesicle (sEV) miRNAs, including miR-200b-3p, miR-200c-3p, miR-10a-5p, miR-18a-3p, miR-95-3p, and miR-3158-3p (Supplementary Fig. 4). In addition, no significant differences were observed in the tissue tumor mutational burden (TMB), PD-L1 expression level, or mutant-allele tumor heterogeneity (MATH) (Supplementary Fig. 2).

To identify the independent factors associated with PFS and OS, multivariate Cox regression analysis was further performed. The results demonstrated that the presence of liver metastases was an independent prognostic factor for poor PFS (HR = 5.66, 95% CI 1.58–20.2) and OS (HR = 7.85, 95% CI 1.38–44.8), whereas FLT mutation was independently associated with poor OS (HR = 12.5, 95% CI 1.54–101) (Supplementary Table 2).

## Discussion

To the best of our knowledge, this is the first trial to prospectively evaluate the combination of immunotherapy with antiangiogenic therapy among mCRC patients in the first-line setting. This trial met the primary endpoint, revealing that first-line sintilimab plus anlotinib treatment offers promising antitumor activity with favorable efficacy, durability, and tolerability in treatment-naïve mCRC patients. This chemotherapy-free regimen yielded an ORR of 48.3%, with a mPFS of 8.6 months, although 50.0% of the enrolled patients had an ECOG score of 2. This combination of sintilimab plus anlotinib also exhibited an acceptable safety profile, with a significantly lower incidence of AEs than those associated with chemotherapy plus anti-EGFR/VEGFR therapy.

The current standard-of-care for mCRC patients involves chemotherapy regimens involving the use of fluorouracil, oxaliplatin, and/or irinotecan combined with agents targeting angiogenesis (bevacizumab) or epidermal growth factor receptor (cetuximab or panitumumab) on the basis of RAS and BRAF status.^26–28^ This triple combination has demonstrated substantial clinical benefits for mCRC patients. For example, the TAILOR trial revealed that the combination of cetuximab with FOLFOX yielded an ORR of 61.1%, along with mPFS and mOS durations of 9.2 and 20.7 months, respectively, among Chinese mCRC patients.^29^ However, despite these significant clinical benefits, adverse events associated with the current standard first-line regimen cannot be ignored. The CALGB/SWOG80405 study reported that 96% of patients experienced at least one adverse event, while 53% of them experienced grade 3 or higher AEs when receiving chemotherapy plus bevacizumab or cetuximab.^4^ The FIRE-3 trial reported an even higher incidence of grade 3 or higher AEs (ranging from 64% to 71%).^30^ Moreover, 15% of patients who received cetuximab in combination with chemotherapy and 11% who received bevacizumab discontinued treatment due to treatment-related toxicity.^30^ In this era of immunotherapy, several prior attempts to combine immunotherapy and chemotherapy in the first-line treatment of mCRC have also been made, resulting in suboptimal efficacy. Instead, a significant increase in the risk of AEs was observed. The AtezoTRIBE study revealed that the addition of atezolizumab to first-line FOLFOXIRI plus bevacizumab might improve PFS in mCRC patients, but 67% of patients experienced grade 3–4 AEs, and 65% required at least one treatment dose reduction due to AEs.^31^ The development of AEs caused by these regimens was a potential reason for patients’ refusal of standard doublet or triplet first-line chemotherapy among mCRC patients, especially patients with poor performance conditions. Notably, sintilimab plus anlotinib had a significant advantage in terms of safety outcomes in this trial. Although our trial recruited more patients with poor health (ECOG 2) than the CALGB/SWOG80405 and FIRE-3 trials did, the rate of grade 3 or higher AEs in our trial was only 13.8%. In addition, modified fluoropyrimidine-based regimens (infusional 5-FU/LV or capecitabine ± bevacizumab) have been recommended for chemotherapy-ineligible mCRC patients. For example, the AVEX phase III trial demonstrated that capecitabine-bevacizumab achieved a mPFS of 9.1 months and a mOS of 20.7 months in elderly patients (≥70 years, 7.5% ECOG PS 2) who were intolerant to intensive chemotherapy, with grade ≥3 adverse events (AEs) in 59% of participants.^32^ In contrast, our chemotherapy-free combination had a superior ORR (48.3% vs. 19.3%) and comparable mOS (22.9 months vs. 20.7 months) despite enrolling higher-risk patients (50% ECOG PS 2). Notably, our novel regimen significantly reduced the incidence of grade ≥ 3 TRAEs (13.8% vs. 59%). Compared with the LCCC1632 trial investigating a chemotherapy-free regimen (panitumumab, ipilimumab, and nivolumab) as a second-line or later regimen among mCRC patients,^33^ superior efficacy and enhanced safety could also be observed in the APICLA-CRC trial cohort. Given the cross-trial, nonrandomized design of these comparisons, the results warrant cautious interpretation and necessitate validation through prospective randomized controlled trials.

As reported in the REGONIVO trial, the combination of antiangiogenic TKI with an anti-PD-1 antibody has been widely investigated among heavily treated mCRC patients worldwide. There were several unfavorable efficacy factors for immunotherapy in our trial, such as poor ECOG-PS (50%) and the presence of liver metastases (63.3%), but the regimen nonetheless provided better efficacy than did previous similar trials. Cumulative evidence has revealed that organ site has a differential influence on the response to immunotherapy, with lymph node, lung, and liver metastasis patients being the most, intermediate, and least responsive, respectively.^34,35^ mCRC patients with liver metastases often had inferior PFS than those without liver metastases when receiving immunotherapy. Mechanistically, the liver often harbors a relatively high proportion of immunosuppressive cells that were responsible for liver-metastasis-associated resistance to immune checkpoint inhibitors,^36,37^ and liver metastasis appears to be correlated with a poor response to antiangiogenic agents.^38^ Consistent with these results, the multivariate Cox regression analysis demonstrated that the presence of liver metastases was an independent prognostic factor for both poor PFS and poor OS in our trial. Notably, patients without hepatic metastasis respond well and benefit more from the combination of sintilimab plus anlotinib, suggesting that they are good candidates for this regimen. In addition, to better reflect the real-world characteristics of patients in a first-line setting, particularly patients who prefer to receive chemotherapy-free regimens due to poor performance scores, our trial recruited patients with an ECOG status of 2 who were excluded from previous trials, such as REGONIVO and REGOTORI.^13,39^ A good PS was correlated with favorable efficacy of immunotherapy or antiangiogenic agents alone.^10,38,40^ Thus, the high proportion of patients with ECOG performance scores of 2 in our trial may also have affected the efficacy of sintilimab plus anlotinib. Another factor is that our trial excluded dMMR/MSI-H CRC patients, who respond well to immunotherapy. Despite these unfavorable factors, our trial still achieved favorable efficacy.

To identify potential efficacy-related biomarkers guiding patient selection and therapeutic optimization for immunotherapy in mCRC, we conducted an exploratory analysis. It has been reported that *RAS*-mutated mCRC patients were associated with an immunosuppressive microenvironment.^41,42^ *KRAS* mutation was independently associated with reduced immune infiltration and reactivity in patients with colorectal cancer,^43^ which may account for the low efficacy of immunotherapy in mCRC patients.^44^ Both the CHECKMATE-8HW and KEYNOTE-177 trials confirmed that mCRC patients with KRAS mutations obtained less benefit from immunotherapy.^33,45^ Consistent with these findings, our data also revealed that *KRAS*-mutated mCRC patients had shorter PFS and OS from sintilimab plus anlotinib than their wild-type counterparts did. *PIK3R1* and *FLT4* mutations were significantly associated with decreased PFS and OS. In addition, previous studies have suggested that *ARID1A* alterations may contribute to impaired MMR or mutation phenotypes in cancer patients and could cooperate with immune checkpoint blockade therapy.^46–48^ Consistent with these reports, our study revealed a positive effect of *ARID1A* mutation on the efficacy of sintilimab plus anlotinib. However, multivariate Cox regression analysis adjusted for confounding factors demonstrated that only the FLT4 mutation was an independent prognostic predictor for poor PFS. In addition, all patients with long PFS—including one patient with an ongoing PFS of 48 months, two patients with PFS longer than 24 months, and five patients with PFS exceeding 12 months—did not have FLT4 mutations. Further biomarker exploration is needed to elucidate the underlying mechanisms of long PFS among patients who received this combination regimen.

In addition, the Checkmate 9 × 8 and AtezoTRIBE trials suggested that the immune score (IC) could be a promising biomarker. The immune score (IC) was high in 32% of pMMR tumors, independently impacting the clinical benefit of immunotherapy as well as chemotherapy.^49^ This signature represents a potential biomarker for selecting pMMR mCRC patients who were likely to benefit from immunotherapy. Our study evaluated the associations of immune-microenvironment characteristics with the efficacy of sintilimab plus anlotinib, which was in line with the immune score (IC). The results demonstrated that mCRC patients who benefit more from sintilimab plus anlotinib often exhibit greater infiltration of immune cells, including PD-1+ CD8+ T cells, PD-L1+ CD68+ macrophages, CD20+ B cells, and CD56dim NK cells, into the tumor microenvironment. Moreover, previous studies reported that immune cell spatial heterogeneity may be associated with cancer development and response to immunotherapy.^50–52^ Consistent with these findings, our spatial analysis revealed that an increased density of PD-1+ cells and CD68+ CD163+ macrophages was localized close to colorectal cancer (<30 μm) in mCRC patients who achieved a clinical response from sintilimab plus anlotinib. In addition, our study was the first to report the association of plasma exosomal miRNAs with the efficacy of sintilimab plus anlotinib in mCRC patients. These differentially expressed miRNAs may be considered biomarkers for predicting the response to this novel first-line regimen.

This study aimed to rapidly evaluate the antitumor potential of this combination in the first-line setting. This trial is a single-institution, single-arm design with ORR as the primary endpoint. Therefore, there are limitations to the current study. First, the lack of a comparator group prevented precise quantification of the treatment effect of this “chemotherapy-free” regimen compared with the standard-of-care. The small number of enrolled patients resulted in wide confidence intervals for PFS and OS, especially in the multivariate Cox regression analysis. Second, strict adherence to ethical requirements and slow screening and enrollment of eligible patients extended the duration of the trial, but this phase II trial validated the efficacy and safety of the therapeutic combination. Furthermore, the study demonstrated a favorable safety profile for this regimen; however, its impact on quality of life (QoL) requires further investigation for validation.

In conclusion, this phase II trial demonstrated that sintilimab plus anlotinib exhibited preliminary antitumor efficacy and a manageable safety profile as a chemotherapy-free approach in treatment-naïve mCRC patients.

## Materials and methods

### Study design and participants

This study was designed as a single-center, open-label, single-arm, phase II trial that recruited treatment-naïve mCRC patients from Shanghai Changzheng Hospital (APICAL-CRC ClinicalTrials.gov number, NCT04271813). The key eligible inclusion criteria were (1) age between 18 and 75 years, with histologically confirmed unresectable and metastatic colorectal cancer (AJCC-TNM stage IV adenocarcinoma of the colon or rectum); (2) absence of prior systematic anticancer treatment, relapse or metastases occurring more than 12 months after adjuvant chemotherapy; (3) presence of at least one measurable lesion as per the Response Evaluation Criteria in Solid Tumors version 1.1 (RECIST 1.1)^53^; (4) Eastern Cooperative Oncology Group (ECOG) performance status of 0–2; (5) adequate organ functions: Hemoglobin ≥ 90 g/L, Absolute Neutrophil Count (ANC) ≥ 1.5 × 10^9^/L, Platelet count ≥ 80 × 10^9^/L, Bilirubin < 1.5 times the upper limit of normal (ULN), Alanine Aminotransferase (ALT) and Aspartate Aminotransferase (AST) < 2.5 × ULN in the absence of liver metastases, and Bilirubin < 3 × ULN, ALT and AST < 5 × ULN in the presence of liver metastases, Serum Creatinine ≤ 1.5 × ULN; (6) completion of radiotherapy at least 3 weeks prior to recruitment, with the caveat that lesions undergoing radiotherapy could not be utilized for clinical benefit assessment using RECIST criteria. The main exclusion criteria were (1) dMMR/MSI-H status; (2) prior anlotinib or immune checkpoint inhibitor treatment; (3) brain metastasis inability to accurately describe the condition; (4) the presence of autoimmune disease or a history of organ transplantation; (5) treatment with glucocorticoids (>10 mg prednisone per day) and immunosuppressive agents; (6) a history of a second malignancy within the past 5 years before inclusion in the study or during participation in the study, with the exception of dermal basal cell or squamous cell carcinoma or cervical carcinoma in situ, if these were treated creatively; (7) myocardial infarction, unstable angina pectoris, Grade III or IV heart failure (NYHA classification); and (8) the presence of a significant concomitant disease, as determined by the investigating physician, that precluded the patient’s participation in the study.

This trial was carried out in accordance with the Declaration of Helsinki and Good Clinical Practice Guidelines, and the study was approved by the institutional review board of the ethics committee of Shanghai Changzheng Hospital (Shanghai, China). Written informed consent was obtained from all patients before any study-related procedures were performed.

### Procedures

Eligible patients were given sintilimab intravenously at a dose of 200 mg on day 1 and anlotinib orally at a dosage of 12 mg daily from days 1–14 every 3 weeks. Treatment was maintained until progressive disease (PD), death, development of intolerable toxicity, or withdrawal of consent. The management of toxicity involved supportive care, as well as prescheduled reductions in the dose of anlotinib and interruptions of the anlotinib dose until adverse events (AEs) were tolerable (grade 2 or lower). Anlotinib could be reduced if patients had intolerable grade 3 or higher treatment-related AEs (TRAEs), as judged by the investigators. The first dose reduction of anlotinib was 10 mg per day, whereas the second dose reduction of anlotinib was 8 mg per day. If patients could still not tolerate 8 mg daily, anlotinib administration was terminated. If the anlotinib dose was reduced or interrupted, subsequent cycles could not include dose increases. Modifications to the sintilimab dosage were not permitted, and sintilimab was temporarily halted for grade 3 or higher immune-related AEs. Patients experiencing intolerable AEs leading to the delay or discontinuation of one medication continued treatment with the other.

### Assessment

Tumor responses were assessed by the investigators via CT (chest, abdomen, and pelvis) or MRI (liver lesion, if necessary after CT scan) scans at study initiation and every 6 weeks until disease progression, in accordance with the RECIST version 1.1 criteria. Eligible patients underwent evaluations, tests, and measurements every 3 weeks, including hematology, serum chemistry, and ECOG performance status. During the treatment period, AEs were assessed according to the National Cancer Institute Common Terminology Criteria for Adverse Events version 5.0.

Before enrollment, formalin-fixed paraffin-embedded (FFPE) tissue from eligible patients was analyzed to determine the baseline molecular characteristics, including mismatch repair (MMR) status, molecular alteration profiles, PD-L1 expression, tumor microenvironment (TME) characteristics, and plasma exosomal miRNAs. PD-L1 immunohistochemistry (22C3 antibody; Agilent Technology, CA, USA) was used to evaluate the PD-L1 tumor proportion score (TPS) by a pathologist, which was defined as the proportion of PD-L1-positive tumor cells relative to the total number of tumor cells multiplied by 100. The molecular alteration profile of eligible patients was assessed via customized next-generation sequencing panels targeting 733 cancer-related genes in FFPE tissues (3D Medicine, Shanghai, China). In addition, plasma exosomal miRNAs were also identified. The TME was evaluated by multiplex immunofluorescence (mIF) using the Akoya OPAL Polaris 7-Color Automation IHC Kit (NEL871001KT). For further details regarding these analytical approaches, see the Supplementary Appendix.

### Outcomes

The primary endpoint was the ORR, which was assessed by the investigators following RECIST version 1.1 guidelines. The secondary endpoints included DCR, PFS, OS, and safety. The ORR was determined on the basis of the number of patients who achieved complete or partial response, while the DCR was considered the best overall response, comprising complete response, partial response, and stable disease. PFS was calculated from the patient’s enrollment date to the point of disease progression or death from any cause, whereas OS was measured from enrollment to the date of death from any cause. In exploratory analyses, efficacy-related biomarkers were further identified by comparing molecular characteristics between responders (CR + PR) and nonresponders (SD + PD).

### Statistical analysis

Sample size estimation was performed via Simon’s minimax two-stage design.^54^ For patients with impaired tolerance to aggressive initial therapy, previous trials have shown that 5-FU/LV or capecitabine with or without bevacizumab achieved an ORR of 19–34.0%.^32,55^ The response rates reported in previous studies involving PD-1 antibodies plus antiangiogenic TKIs in heavily treated Chinese mCRC patients ranged from 15.2% (regorafenib plus toripalimab) to 23.6% (sintilimab plus fruquintinib).^25,39^ The combination of regorafenib plus nivolumab yielded an ORR of 33% in MSS mCRC patients.^13^ Therefore, we anticipated that the combination of sintilimab plus anlotinib would achieve an improved ORR of 35%. The null hypothesis postulated a true ORR of 15%, which was tested against a one-sided alternative of 35%, with 80% power and a type I error rate of 5%. In the first stage of this study, 15 patients were planned to be recruited. If fewer than two responses were observed, the study was terminated and deemed negative. Otherwise, another 13 patients would be recruited for the second stage, and the study would be considered positive if more than 9 responders were observed among the 28 patients.

A descriptive summary of patient characteristics, safety data, and antitumor activity was provided. The ORR and DCR 95% confidence intervals (CIs) were determined via the Clopper and Pearson methods, and the Kaplan–Meier method was used to estimate PFS and OS. Patients who received at least one radiological assessment were evaluated for activity endpoints for the efficacy-evaluable cohort, whereas safety endpoints were analyzed for patients who received protocol treatment regardless of eligibility in the intent-to-treat (ITT) cohort. Fisher’s exact test was used for prespecified exploratory analyses to compare the ORR and other binary outcomes among different subgroups. All the statistical analyses were conducted via R software (https://www.r-project.org/, v 20.0.3; Belgium).

## Supplementary information

Trial-Protocol

Supplementary Materials

## Data Availability

All sequencing data generated in this study have been deposited in the National Genomics Data Center (NGDC) under the project accession number PRJCA042908, which is accessible at https://ngdc.cncb.ac.cn/bioproject/. All the data generated or analyzed during this study are available from the corresponding authors.

## References

[1] Sung, H. et al. Global Cancer Statistics 2020: GLOBOCAN estimates of incidence and mortality worldwide for 36 cancers in 185 countries. *CA Cancer J. Clin.* **71**, 209–249 (2021). 33538338 10.3322/caac.21660

[2] Cervantes, A. et al. Metastatic colorectal cancer: ESMO Clinical Practice Guideline for diagnosis, treatment and follow-up. *Ann. Oncol.* **34**, 10–32 (2023). 36307056 10.1016/j.annonc.2022.10.003

[3] Tejpar, S. et al. Prognostic and predictive relevance of primary tumor location in patients with RAS wild-type metastatic colorectal cancer: retrospective analyses of the CRYSTAL and FIRE-3 trials. *JAMA Oncol.* **3**, 194–201 (2017). 27722750 10.1001/jamaoncol.2016.3797 PMC7505121

[4] Venook, A. P. et al. Effect of first-line chemotherapy combined with cetuximab or bevacizumab on overall survival in patients with KRAS wild-type advanced or metastatic colorectal cancer: a randomized clinical trial. *JAMA* **317**, 2392–2401 (2017). 28632865 10.1001/jama.2017.7105 PMC5545896

[5] Andre, T. et al. Trifluridine-tipiracil plus bevacizumab versus capecitabine plus bevacizumab as first-line treatment for patients with metastatic colorectal cancer ineligible for intensive therapy (SOLSTICE): a randomised, open-label phase 3 study. *Lancet Gastroenterol. Hepatol.* **8**, 133–144 (2023). 36470291 10.1016/S2468-1253(22)00334-X

[6] Andre, T. et al. Health-related quality of life in patients with microsatellite instability-high or mismatch repair deficient metastatic colorectal cancer treated with first-line pembrolizumab versus chemotherapy (KEYNOTE-177): an open-label, randomised, phase 3 trial. *Lancet Oncol.* **22**, 665–677 (2021). 33812497 10.1016/S1470-2045(21)00064-4

[7] Andre, T. et al. Pembrolizumab in microsatellite-instability-high advanced colorectal cancer. *N. Engl. J. Med.* **383**, 2207–2218 (2020). 33264544 10.1056/NEJMoa2017699

[8] Diaz, L. A. Jr. et al. Pembrolizumab versus chemotherapy for microsatellite instability-high or mismatch repair-deficient metastatic colorectal cancer (KEYNOTE-177): final analysis of a randomised, open-label, phase 3 study. *Lancet Oncol.* **23**, 659–670 (2022). 35427471 10.1016/S1470-2045(22)00197-8 PMC9533375

[9] Le, D. T. et al. PD-1 blockade in tumors with mismatch-repair deficiency. *N. Engl. J. Med.* **372**, 2509–2520 (2015). 26028255 10.1056/NEJMoa1500596 PMC4481136

[10] Marmorino, F., Boccaccino, A., Germani, M. M., Falcone, A. & Cremolini, C. Immune checkpoint inhibitors in pmmr metastatic colorectal cancer: a tough challenge. *Cancers (Basel)* **12**, 2317 (2020). 32824490 10.3390/cancers12082317 PMC7465130

[11] Fukumura, D., Kloepper, J., Amoozgar, Z., Duda, D. G. & Jain, R. K. Enhancing cancer immunotherapy using antiangiogenics: opportunities and challenges. *Nat. Rev. Clin. Oncol.* **15**, 325–340 (2018). 29508855 10.1038/nrclinonc.2018.29 PMC5921900

[12] Tian, L. et al. Mutual regulation of tumour vessel normalization and immunostimulatory reprogramming. *Nature* **544**, 250–254 (2017). 28371798 10.1038/nature21724 PMC5788037

[13] Fukuoka, S. et al. Regorafenib plus nivolumab in patients with advanced gastric or colorectal cancer: an open-label, dose-escalation, and dose-expansion Phase Ib Trial (REGONIVO, EPOC1603). *J. Clin. Oncol.* **38**, 2053–2061 (2020). 32343640 10.1200/JCO.19.03296

[14] Kawazoe, A. et al. Lenvatinib plus pembrolizumab versus standard of care for previously treated metastatic colorectal cancer: final analysis of the randomized, open-label, Phase III LEAP-017 study. *J. Clin. Oncol.* **42**, 2918–2927 (2024). 38833658 10.1200/JCO.23.02736 PMC11328923

[15] Chi, Y. et al. Safety and efficacy of anlotinib, a multikinase angiogenesis inhibitor, in patients with refractory metastatic soft-tissue sarcoma. *Clin. Cancer Res* **24**, 5233–5238 (2018). 29895706 10.1158/1078-0432.CCR-17-3766

[16] Han, B. et al. Effect of anlotinib as a third-line or further treatment on overall survival of patients with advanced non-small cell lung cancer: the ALTER 0303 Phase 3 randomized clinical trial. *JAMA Oncol.* **4**, 1569–1575 (2018). 30098152 10.1001/jamaoncol.2018.3039 PMC6248083

[17] Li, D. et al. Anlotinib in locally advanced or metastatic medullary thyroid carcinoma: a randomized, double-blind Phase IIB Trial. *Clin. Cancer Res.* **27**, 3567–3575 (2021). 33832949 10.1158/1078-0432.CCR-20-2950

[18] Chi, Y. et al. Anlotinib monotherapy for refractory metastatic colorectal cancer: a double-blinded, placebo-controlled, randomized Phase III Trial (ALTER0703). *Oncologist* **26**, e1693–e1703 (2021). 34105207 10.1002/onco.13857 PMC8488800

[19] Liu, Y. et al. Phase II study of anlotinib in combination with oxaliplatin and capecitabine for patients with RAS/BRAF wild-type metastatic colorectal adenocarcinoma as the first-line therapy. *BMC Med.* **20**, 155 (2022). 35513832 10.1186/s12916-022-02357-6 PMC9071922

[20] Bai, H., Wang, W. H., Zhou, F. F., Yang, D. & Li, R. J. Feasibility and tolerability of anlotinib plus PD-1 blockades for patients with treatment-refractory metastatic colorectal cancer: a retrospective exploratory study. *Cancer Manag. Res.* **16**, 73–86 (2024). 38318097 10.2147/CMAR.S427680 PMC10840531

[21] Lu, S. et al. Sintilimab plus bevacizumab biosimilar IBI305 and chemotherapy for patients with EGFR-mutated non-squamous non-small-cell lung cancer who progressed on EGFR tyrosine-kinase inhibitor therapy (ORIENT-31): first interim results from a randomised, double-blind, multicentre, phase 3 trial. *Lancet Oncol.* **23**, 1167–1179 (2022). 35908558 10.1016/S1470-2045(22)00382-5

[22] Ren, Z. et al. Sintilimab plus a bevacizumab biosimilar (IBI305) versus sorafenib in unresectable hepatocellular carcinoma (ORIENT-32): a randomised, open-label, phase 2-3 study. *Lancet Oncol.* **22**, 977–990 (2021). 34143971 10.1016/S1470-2045(21)00252-7

[23] Chu, T. et al. Phase 1b study of sintilimab plus anlotinib as first-line therapy in patients with advanced NSCLC. *J. Thorac. Oncol.* **16**, 643–652 (2021). 33524601 10.1016/j.jtho.2020.11.026

[24] Xu, Q. et al. Efficacy and safety of sintilimab plus anlotinib for PD-L1-positive recurrent or metastatic cervical cancer: a Multicenter, Single-Arm, Prospective Phase II Trial. *J. Clin. Oncol.* **40**, 1795–1805 (2022). 35192397 10.1200/JCO.21.02091 PMC9148684

[25] Guo, Y. et al. Phase 1b/2 trial of fruquintinib plus sintilimab in treating advanced solid tumors: the dose-escalation and metastatic colorectal cancer cohort in the dose-expansion phases. *Eur. J. Cancer* **181**, 26–37 (2023). 36628898 10.1016/j.ejca.2022.12.004

[26] Benson, A. B. et al. Colon cancer, version 2.2021, NCCN clinical practice guidelines in oncology. *J. Natl Compr. Cancer Netw.* **19**, 329–359 (2021). 10.6004/jnccn.2021.0012 33724754

[27] Diagnosis & Treatment Guidelines For Colorectal Cancer Working Group, C Chinese Society of Clinical Oncology (CSCO) diagnosis and treatment guidelines for colorectal cancer 2018 (English version). Chin. J. Cancer Res. 31, 117–134 (2019). 10.21147/j.issn.1000-9604.2019.01.07 PMC6433585 30996570

[28] Rossini, D. et al. Upfront modified fluorouracil, leucovorin, oxaliplatin, and irinotecan plus panitumumab versus fluorouracil, leucovorin, and oxaliplatin plus panitumumab for patients with RAS/BRAF wild-type metastatic colorectal cancer: the Phase III TRIPLETE study by GONO. *J. Clin. Oncol.* **40**, 2878–2888 (2022). 35666229 10.1200/JCO.22.00839 PMC9426812

[29] Qin, S. et al. Efficacy and tolerability of first-line cetuximab plus leucovorin, fluorouracil, and oxaliplatin (FOLFOX-4) versus FOLFOX-4 in patients with RAS wild-type metastatic colorectal cancer: the open-label, randomized, Phase III TAILOR trial. *J. Clin. Oncol.* **36**, 3031–3039 (2018). 30199311 10.1200/JCO.2018.78.3183 PMC6324088

[30] Heinemann, V. et al. FOLFIRI plus cetuximab versus FOLFIRI plus bevacizumab as first-line treatment for patients with metastatic colorectal cancer (FIRE-3): a randomised, open-label, phase 3 trial. *Lancet Oncol.* **15**, 1065–1075 (2014). 25088940 10.1016/S1470-2045(14)70330-4

[31] Antoniotti, C. et al. Upfront FOLFOXIRI plus bevacizumab with or without atezolizumab in the treatment of patients with metastatic colorectal cancer (AtezoTRIBE): a multicenter, open-label, randomised, controlled, phase 2 trial. *Lancet Oncol.* **23**, 876–887 (2022). 35636444 10.1016/S1470-2045(22)00274-1

[32] Cunningham, D. et al. Bevacizumab plus capecitabine versus capecitabine alone in elderly patients with previously untreated metastatic colorectal cancer (AVEX): an open-label, randomised phase 3 trial. *Lancet Oncol.* **14**, 1077–1085 (2013). 24028813 10.1016/S1470-2045(13)70154-2

[33] Lee, M. S. et al. Phase II study of ipilimumab, nivolumab, and panitumumab in patients with KRAS/NRAS/BRAF wild-type (WT) microsatellite stable (MSS) metastatic colorectal cancer (mCRC). *J. Clin. Oncol.* **39**, (XX,_Suppl. 7) (2021).

[34] Osorio, J. C. et al. Lesion-level response dynamics to programmed cell death protein (PD-1) blockade. *J. Clin. Oncol.* **37**, 3546–3555 (2019). 31675272 10.1200/JCO.19.00709 PMC7194449

[35] Pires da Silva, I. et al. Site-specific response patterns, pseudoprogression, and acquired resistance in patients with melanoma treated with ipilimumab combined with anti-PD-1 therapy. *Cancer* **126**, 86–97 (2020). 31584722 10.1002/cncr.32522

[36] Lindblad, K. E. & Lujambio, A. Liver metastases inhibit immunotherapy efficacy. *Nat. Med.* **27**, 25–27 (2021). 33442002 10.1038/s41591-020-01190-9

[37] Yu, J. et al. Liver metastasis restrains immunotherapy efficacy via macrophage-mediated T cell elimination. *Nat. Med.* **27**, 152–164 (2021). 33398162 10.1038/s41591-020-1131-x PMC8095049

[38] Martinelli, E. et al. Clinical outcome and molecular characterisation of chemorefractory metastatic colorectal cancer patients with long-term efficacy of regorafenib treatment. *ESMO Open* **2**, e000177 (2017). 29211816 10.1136/esmoopen-2017-000177 PMC5703385

[39] Wang, F. et al. Regorafenib plus toripalimab in patients with metastatic colorectal cancer: a phase Ib/II clinical trial and gut microbiome analysis. *Cell Rep. Med.* **2**, 100383 (2021). 34622226 10.1016/j.xcrm.2021.100383 PMC8484502

[40] Grothey, A. et al. Regorafenib monotherapy for previously treated metastatic colorectal cancer (CORRECT): an international, multicentre, randomised, placebo-controlled, phase 3 trial. *Lancet* **381**, 303–312 (2013). 23177514 10.1016/S0140-6736(12)61900-X

[41] Patelli, G. et al. Strategies to tackle RAS-mutated metastatic colorectal cancer. *ESMO Open* **6**, 100156 (2021). 34044286 10.1016/j.esmoop.2021.100156 PMC8167159

[42] Modest, D. P. et al. Outcome according to KRAS-, NRAS- and BRAF-mutation as well as KRAS mutation variants: pooled analysis of five randomized trials in metastatic colorectal cancer by the AIO colorectal cancer study group. *Ann. Oncol.* **27**, 1746–1753 (2016). 27358379 10.1093/annonc/mdw261 PMC4999563

[43] Lal, N. et al. KRAS mutation and consensus molecular subtypes 2 and 3 are independently associated with reduced immune infiltration and reactivity in colorectal cancer. *Clin. Cancer Res.* **24**, 224–233 (2018). 29061646 10.1158/1078-0432.CCR-17-1090 PMC5777581

[44] Liao, W. et al. KRAS-IRF2 axis drives immune suppression and immune therapy resistance in colorectal cancer. *Cancer Cell* **35**, 559–572 e557 (2019). 30905761 10.1016/j.ccell.2019.02.008 PMC6467776

[45] André, T. et al. Nivolumab plus ipilimumab versus nivolumab in microsatellite instability-high metastatic colorectal cancer (CheckMate 8HW): a randomised, open-label, phase 3 trial. *Lancet* **405**, 383–395 (2025). 39874977 10.1016/S0140-6736(24)02848-4

[46] Okamura, R. et al. ARID1A alterations function as a biomarker for longer progression-free survival after anti-PD-1/PD-L1 immunotherapy. *J. Immunother. Cancer* **8**, e000438 (2020). 32111729 10.1136/jitc-2019-000438 PMC7057434

[47] Shen, J. et al. ARID1A deficiency promotes mutability and potentiates therapeutic antitumor immunity unleashed by immune checkpoint blockade. *Nat. Med.* **24**, 556–562 (2018). 29736026 10.1038/s41591-018-0012-z PMC6076433

[48] Mehrvarz Sarshekeh, A. et al. ARID1A mutation may define an immunologically active subgroup in patients with microsatellite stable colorectal cancer. *Clin. Cancer Res.* **27**, 1663–1670 (2021). 33414133 10.1158/1078-0432.CCR-20-2404 PMC7956157

[49] Pages, F. et al. Prognostic and predictive value of the Immunoscore in stage III colon cancer patients treated with oxaliplatin in the prospective IDEA France PRODIGE-GERCOR cohort study. *Ann. Oncol.* **31**, 921–929 (2020). 32294529 10.1016/j.annonc.2020.03.310

[50] Attrill, G. H. et al. Higher proportions of CD39+ tumor-resident cytotoxic T cells predict recurrence-free survival in patients with stage III melanoma treated with adjuvant immunotherapy. *J. Immunother. Cancer* **10**, e004771 (2022). 35688560 10.1136/jitc-2022-004771 PMC9189855

[51] Cheung, P. F. et al. Progranulin mediates immune evasion of pancreatic ductal adenocarcinoma through regulation of MHCI expression. *Nat. Commun.* **13**, 156 (2022). 35013174 10.1038/s41467-021-27088-9 PMC8748938

[52] Huang, Y. K. et al. Macrophage spatial heterogeneity in gastric cancer defined by multiplex immunohistochemistry. *Nat. Commun.* **10**, 3928 (2019). 31477692 10.1038/s41467-019-11788-4 PMC6718690

[53] Eisenhauer, E. A. et al. New response evaluation criteria in solid tumours: revised RECIST guideline (version 1.1). *Eur. J. Cancer* **45**, 228–247 (2009). 19097774 10.1016/j.ejca.2008.10.026

[54] Simon, R. Optimal two-stage designs for phase II clinical trials. *Control Clin. Trials* **10**, 1–10 (1989). 2702835 10.1016/0197-2456(89)90015-9

[55] Kabbinavar, F. F. et al. Combined analysis of efficacy: the addition of bevacizumab to fluorouracil/leucovorin improves survival for patients with metastatic colorectal cancer. *J. Clin. Oncol.* **23**, 3706–3712 (2005). 15867200 10.1200/JCO.2005.00.232

