# Supplementary material for: Anlotinib plus sintilimab as first-line treatment for patients with advanced colorectal cancer (APICAL-CRC): an open-label, single-arm, phase II trial
Source: Signal Transduct Target Ther. 2025 Sep 16;10:301. doi: 10.1038/s41392-025-02383-9 (PMC12436658; doi:10.1038/s41392-025-02383-9)
Supplement: Supplementary file 1 — Trial-Protocol [file 41392_2025_2383_MOESM1_ESM.docx]

**Anlotinib plus Sintilimab as first-line treatment for patients with advanced colorectal cancer (APICAL-CRC): a single center, open-label, single arm, phase II study**

**Investigator-initiated Study Protocol**

**Protocol Number**: **APICAL-CRC**

Primary Investigator： Yuan-Sheng Zang

Department of Medical Oncology, Shanghai Changzheng Hospital, Naval Military Medical University, China

64 Hetian Road, Shanghai, China

Tel: 86-02166540109-8002

E-mail: [doctorzangys@163.com](mailto:doctorzangys@163.com)

**0. SYNOPSIS**

| Study Title | Anlotinib plus Sintilimab as first-line treatment for patients with advanced colorectal cancer (APICAL-CRC): a single center, open-label, single arm, phase II study |
| --- | --- |
| Phase | Phase II |
| Objectives | To assess the safety and efficacy of a new regimen using Anlotinib plus Sintilimab as first-line treatment for patients with advanced colorectal cancer |
| Study Endpoint | Primary endpoint：  Objective response rate (ORR) by Response Evaluation Criteria in Solid Tumors (RECIST) ver1.1  Secondary endpoints:  ・Efficacy  - Disease control rate (DCR)  - Progression-free survival (PFS)  - Overall survival (OS)  ・Safety  - The incidences and types of adverse events that occur during treatment will be evaluated according to the National Cancer Institute (NCI) Common Terminology Criteria for Adverse Events (CTCAE) version 5.0.  Exploration biomarker analysis:  PD-L1 expression, TMB, molecular alteration profile, tumor immune-microenvironment, plasma exosome miRNA. |
| Study Population | According to the inclusion criteria, all mCRC patients required to be treatment-naïve, unresectable and metastatic CRCs.  **Inclusion criteria**   - Patients have histologically or cytologically confirmed advanced or recurrent CRC; - No prior systematic anti-cancer treatment and relapse or metastases was occurred more than 12 months after adjuvant chemotherapy; - Patients have measurable disease as defined by RECIST 1.1 as determined by investigator; - Patient with a history of radiotherapy at least 3 months before on the day of providing consent, but the measurable lesion should not be within the scope of radiotherapy; - Patients with age of 18-75yr; - Patients with a performance status of 0,1or 2 on the Eastern Cooperative Oncology Group.； - Patients with Life expectancy of more than 12 weeks; - Patients must have the ability to understand and sign the written informed consent voluntarily; - Female of childbearing potential who are negative in a pregnancy test within 7 days before enrollment. Both male and female patients should agree to use an adequate method of contraception (total abstinence, an intrauterine device or hormone releasing system, a contraceptive implant and an oral contraceptive) starting with the first dose of study therapy through 120 days after the last dose of study therapy. Duration will be determined when the subject is assigned to treatment.   **Exclusion criteria**   - Patients with dMMR/MSI-H; - Patients with major surgery or severe trauma within 4 weeks before the first medication; - Patients with hypersensitivity to the components in the study protocol;； - Patients who are ready to give birth or are pregnant; - Patients with brain metastases who are unable to accurately describe the condition; - Patients received immune-suppressive drugs 2 weeks before initial treatment (inhaled cortisol or other steroid hormones≤10 mg/day prednisone or equivalent pharmacophysiologic doses were excluded); - Planned live attenuated vaccine within 4 weeks prior to or during study treatment; - Patients have received anlotinib or anti-PD-1 monoclonal antibody therapy or other therapies that act on T-cell co-stimulation targets or checkpoints; - Within 6 months prior to the start of study treatment, the following diseases appeared: myocardial infarction, severe/unstable angina, NYHA grade 2 or above congestive heart failure, poorly controlled arrhythmias, etc; - Active hepatitis; - Bone marrow, liver and kidney function did not meet the requirements of chemotherapy as follows:   - Neutrophil count<1,500/mm3;  - Platelet count <80,000/mm3;  - Total bilirubin >1.5-times the upper limit of normal;  -ALT/AST>2.5-times the upper limit of normal for patients without liver  metastases; (5.0-times the upper limit of normal for patients with  liver metastases)  - Creatinine >1.5-times the upper limit of normal;   - Patients with cancers other than advanced colorectal cancer within five years prior to the start of treatment in this study. Cervical carcinoma in situ, cured basal cell carcinoma and bladder epithelial tumor were excluded; - History of substance abuse, drug use, alcohol dependence; - Patients without legal capacity or limited civil capacity; - Patients with autoimmune diseases or organ transplantation; - Other situations that the investigator deemed inappropriate for enrollment; |
| Duration of study participation | The investigational drug will be repeatedly administered until the criteria for discontinuation are met. In the event that the treatment is discontinued for reasons other than an exacerbation of the underlying disease via imaging (e.g., unacceptable side effects), tumor evaluation follow-up will continue until either an exacerbation of the underlying disease is observed via imaging or a new anticancer treatment is initiated, whichever happens first. |
| Administration method | Eligible patients were given sintilimab intravenously at a dose of 200mg on day 1, and anlotinib orally at a dosage of 12mg daily from days 1-14 every 3 weeks. Treatment was maintained until progressive disease (PD), treatment intolerance, death, or withdrawal of consent, with one cycle as 3 weeks. |
| Safety evaluation | Evaluations will be performed using CTCAE version 5.0. |
| Efficacy evaluation | The efficacy will be evaluated in accordance with RECIST version 1.1 every 6 weeks.  ・Image evaluation: computed tomography (CT) or magnetic resonance imaging (MRI) |
| Observations and tests | Physical examination, vital signs, PS, laboratory tests (hematology and biochemistry), urinalysis, tumor markers, ECG, CT of chest, abdomen, and pelvis, etc. |
| Trial Design | APICAL-CRC trial was an investigator-initiated, open-label, non-randomized, single-arm, phase II clinical trial using Simon’s minimax two-stage design. The combination of anlotinib plus sintilimab is expected to result in an improved ORR of 35%. The null hypothesis, which stated that the true ORR was 15%, would be tested against a one-sided alternative of 35%, with 80% power and a type I error rate of 5%. In the first stage, the study accrued 15 patients. If less than two response or no response was observed, the study was terminated and deemed negative. Otherwise, an additional 13 patients would be accrued for the second stage. The study was considered positive if there were more than 9 responders among the 28 patients. |
| Statistical matters | Primary endpoint (In the intention-to-treat population and in the efficacy-evaluable):  The statistical criteria for main analysis will be based on the number of patients with the best overall response of Complete Response (CR) or Partial Response (PR) according to RECIST guideline version 1.1. An accurate confidence interval based on a binomial distribution is used for calculating the proportion of confidence intervals. ORR by RECIST 1.1  Secondary endpoint  The following analyses will be performed for ITT (Intention to Treat: ITT) and efficacy-evaluable population, SP (Safety Population).   1. DCR by RECIST 1.1(In the intention-to-treat population and in the efficacy-evaluable population): Confidence intervals for DCR will be created using the exact test according to a binomial distribution. 2. PFS, OS: A survival function will be estimated using the Kaplan-Meier method. The median with the 95% CI will be estimated. 3. Incidence of adverse events: As incidence of adverse events, in addition to tabulation of the frequency of adverse events, the incidence of Grade 3 or higher adverse events will be calculated. |
| Primary investigator | Yuan-Sheng Zang |
| Planned study sites | Shanghai Changzheng Hospital |

Contents

**1.Introduciton 4**

1.1 advanced CRC 4

1.2 Treatment Strategy for advanced CRC 4

1.3 Immune checkpoint inhibitor and advanced CRC 4

**2.Objective 7**

**3.Method 7**

3.1 Study Design 7

3.2 Single-arm 8

3.3 Participants 8

3.3.1 Inclusion criteria 8

3.3.2 Exclusion criteria 8

3.4 Study time 10

3.5 Withdrawn from the study 10

3.6 Procedures 10

3.6.1 The use of medication 10

3.6.2 Dose delay and dose adjustment of medication 11

3.6.2.1 Dose adjustment of medication 11

3.6.2.2 Laboratory test before the next cycle of chemotherapy 11

3.6.2.3 Dose adjustment plan for next cycle 12

3.6.3 Concomitant treatment 12

3.6.3.1 Unallowed Treatment 12

3.6.3.2 Allowed Treatment 12

3.6.4 Treatment compliance 13

3.7 Study Endpoint 13

3.7.1 Efficacy Evaluation 13

3.7.2 Safety 13

3.7.3 Starting Time of Study 14

3.7.4.1 Treatment Period 14

3.7.4.2 Imaging Evaluation 14

3.8 Trial Phase 14

3.8.1 Screening Phase 14

3.8.2 Treatment Phase 15

3.8.3 At the end of the study 15

3.8.4 Temporary Visit 15

3.8.5 Follow-up After Disease Progression 15

3.9 Data Quality 16

3.10 Data File 16

**4. Ethical and Legal Issues 16**

4.1 Ethics Committee or Institutional Review Board 16

4.2 The ethical guidance of the study 16

4.3 Subject information and informed consent 17

4.4 Secrecy 17

**5. Statistical Methods 17**

5.1 Statistics analysis plan 17

5.1.1 The population for analysis 17

5.1.2 Baseline and demographic characteristics 17

5.1.3 Efficacy Analysis 17

5.1.3.1 Primary Study Endpoint 17

5.1.3.2 Secondary Study Endpoint 18

5.1.3.3 Exploration biomarker analysis 18

5.1.3.4 Safety Analysis 18

**6. Adverse Events 18**

6.1 Detection of adverse events 18

6.2 Definition of adverse events 18

6.2.1 Adverse events 18

6.2.2 Serious Adverse events 19

6.2.3 Unexpected adverse events 20

6.2.4 Relationship between adverse events and research drugs 20

6.2.5 Record of adverse events 21

6.3 Report of serious adverse events 21

6.4 Prevention and treatment of diarrhea caused by irinotecan 21

**7.** **Appendix 21**

7.1 ECOG 21

7.2 AJCC-TNM staging 22

7.3 RECIST Version 1.1 24

7.4 Flow-chart of the study 26

**8.Reference 27**

1. **Introduction**

**1.1 Advanced Colorectal Cancer**

Colorectal cancer (CRC) ranks the third in terms of cancer incidence and is also the most common malignant tumors in the digestive system, which is a serious threat to human health[1]。In China, approximately 380,000 colorectal cancer cases were newly diagnosed and 200,000 cases dead annually. The incidence and mortality of colorectal cancer patients in city area are higher than that of rural area. For example, colorectal cancer ranks the second in terms of cancer incidence in Shanghai. Almost 1/4 patients were developed advanced stage at the time of diagnosed, half of CRC patients could develop recurrence and distant metastasis, resulting in poor prognoses and short survival[2-3]。

**1.2 Treatment Strategy for advanced CRC**

For advanced or metastatic CRC, chemotherapy is one of the main treatment methods, which plays an important role in controlling the disease, prolonging the survival period and improving the quality of life. There are three commonly used chemotherapy drugs: fluorouracil, oxaliplatin and irinotecan. The researches have not made breakthrough progress on other chemotherapy drugs. In addition, targeted therapy also plays an important role in the treatment of advanced CRC. At present, the combination of chemotherapy and targeted therapy is the standard-of-care recommended by NCCN guidelines [3-5]. Recently, the rise of immune checkpoint blockade inhibitor provides a new field and direction for immunotherapy among advanced CRC patients.

**1.3 Immune checkpoint inhibitor and CRC**

Immune checkpoint inhibitor is currently a hot spot in the treatment of malignant tumors, and has achieved remarkable curative effects in lung cancer, liver cancer, urinary tract malignant tumors, malignant melanoma and hematologic malignant tumors. Immunotherapy has greatly improved the prognosis of cancer patients. Besides, immune checkpoint therapy is the first treatment regimen for pan-tumor approved by the FDA based on molecular characteristics (dMMR/MSI-H).

Based on KEYNOTE016 and CHECKMATE142 studies, pembrolizumab and nivolumab-based immunotherapy regimens have been recommended for the treatment of advanced colorectal cancer with dMMR/MSI-H, especially in patients resistant to chemotherapy plus anti-EGFR/VEGF targeted therapy, with higher recommended level [6]. Currently, the main dominant population of immune checkpoint therapy was advanced CRC patients with dMMR/MSI-H. However, dMMR/MSI-H colorectal cancer only accounts for 3~5% of all advanced CRC patients, and the vast majority of pMMR/MSS patients cannot obtain clinical benefit from immunotherapy alone. In order to change this treatment dilemma, many clinical attempts have been made. For example, the combinations of immunotherapy with other therapeutic means (such as small molecule TKI, chemotherapy, radiotherapy, etc.) were explored to promote the sensitivity to immunotherapy among this “Cold tumor”. Unfortunately, most above trials have failed. The Phase-II trial showed that 8% of advanced CRC patients could achieved PR after received Atezolizumab combined with cobimetinib, with a DCR of 31%. The results make researchers expect the efficacy of subsequent phase III study (IMblaze-370). However, IMblaze-370 did not reach primary endpoints, showing whether Atezolizumab alone or Atezolizumab combined with cobimetinib could not bring significantly more clinical benefit than regorafenib, with the mOS of 7.1m, 8.9m, and 8.5m, respectively [7]. Another phase II study enrolling 30 treatment-naïve advanced CRC patients who received Pembrolizumab combined with mFOLFOX6 observed 1 patient with CR, 15 patients with PR. The efficacy of this combination regimen seems comparable, but not superior to conventional chemotherapy plus targeted therapy. More importantly, 2 out of 6 patients develop G3 granulomyle deficiency fever and 1 G4 neutropenia in safety dose exploratory analysis. MODUL study explored whether the addition of Atezolizumab to conventional fluorouracil plus bevacizumab as a maintenance regimen could bring more clinical benefit. After 18.7 months of follow-up, there was no significant difference between the two groups in either PFS or OS, and the addition of Atezolizumab to standard FP/bevacizumab first-line maintenance regimen did not improve PFS and OS in advanced mCRC patients.

All in all, immunotherapy has limited benefit among patients with advanced colorectal cancer, particularly in the pMMR/MSS cohort. In order to mutate this shackle, researchers have carried out unremitting exploration. A study published in by 2019 ASCO-GI seems to give us a glimmer of light, in which the use of durvalumab (PD-L1 antibody) plus tremelimumab (CTLA-4 antagonist) versus best supportive care (BSC) could prolong OS in patients with advanced colorectal cancer who did not respond to existing treatments. This is the first study in which immunotherapy could significantly more benefit for colorectal cancer patients who have not undergone MMR screening. However, even using dual immunotherapy, mOS duration was only 6.6 months, which was only 2.5 months longer than that of the BSC alone, which is of little significance for clinical practice.

At the ASCO meeting in June 2019, the REGONIVO study rekindled hope for immune checkpoint treatment for advanced colorectal cancer with pMMR/MSS. The REGONIVO study was initially conducted in multiple advanced gastrointestinal tumors, including colorectal cancer, gastric cancer, and liver cancer, with regorafenib dose-ramped in the 2 drugs used and nivolumab using a fixed 3 mg/kg q2w. Efficacy data on the colorectal cancer and gastric cancer showed an overall ORR of 40% in patients who underwent multiple lines of therapy, and an ORR of 36%, 44% for colorectal cancer, and gastric cancer, respectively. More importantly, 33% of pMMR/MSS CRC patients achieved CR or PR. This efficacy seems to be limited, but the significance of the REGONIVO study is significant compared to regorafenib monotherapy in the CORRECT and CONCUR trials with ORRs of 1%, 4% in CORRECT and CONCUR trials, respectively [8, 9]. The ORR of REGONIVO study also was better that of nivolumab in CHECKMATE142 trial, which also had only 10 % ORR in MSS. The PFS of REGONIVO study was 6.3 months in colorectal cancer, which was also a considerable advantage when compared with 1.9 months, 3.2 months in the CORRECT and CONCUR trials. In REGONIVO trial, almost all CRCs patients had received antiangiogenic therapy, and the patients could achieve objective remission regardless of RAS wild type, RAS mutant type, PD-L1 positive or negative expression, indicating that the promising prospect of REGONIVO regimen.

In the preclinical experiment of REGONIVO trial [10], the researchers found that regorafenib could inhibit colony-stimulating factor receptor (CSF-1R) and reduce F4/80+ immune cells in CT26CRC tumors of immunocompromised nude mice growing, suggesting immunomodulatory effects related to antitumor activity. Moreover, regorafenib combined with anti-PD-1 showed a more significant synergistic anti-tumor effect in in vitro experiments. These preliminary studies provide a theoretical basis and support for clinical translational research, suggesting that it is possible that regorafenib increased tumor sensitivity to immune checkpoint therapy. Other small molecule multi-target TKI inhibitors also have similar effects and have been validated in other tumors. A clinical trial using anlotinib combined with sintilimab (anti-PD-1 monoclonal antibody) in the first-line treatment of advanced non-small cell lung cancer, provided an objective response rate of 72.2% and a disease control rate of 100%. This study also enhances us that the immunotherapy-based regimen moves to the front-line could bring more efficacy, because the efficacy of immunotherapy is closely related to the integrity of immune function. Although REGONIVO trial has achieved great success in the treatment of advanced colorectal cancer, only 1/3 patients have achieved objective response, which may be related to late-line use. The first-line treatment effect of advanced colorectal cancer is significantly higher than that of late-line, so we consider that the appropriate regimen in advanced first-line patients may achieve better clinical efficacy and avoid the adverse effects of chemotherapy. We design this trial to explore the efficacy of small molecule multi-target TKI inhibitors (Anlotinib) combined with anti-PD-1 antibodies (Sintilimab) as the first-line regimen for advanced colorectal cancer.

1. **Objective**

To assess the safety and activity of Anlotinib in combination with Sintilimab among treatment-naïve advanced CRC patients.

Primary endpoint：Objective Response Rate (ORR, complete or partial response according to RECIST version 1.1).

Secondary study endpoints: Disease Control Rate (DCR, the proportion of patients who achieved a complete response, a partial response, or stable disease), Progression-Free survival (PFS), overall survival (OS) as well as safety. PFS was defined as the time from therapy initiation to progression according to RECIST. OS is defined as the time from therapy initiation to death from any cause.

Exploration study endpoints: biomarker analysis including PD-L1 expression, TMB, molecular alteration profile, tumor immune-microenvironment, plasma exosome miRNA.

1. **Method**
   1. **Study design**

APICAL-CRC trial was an investigator-initiated, open-label, non-randomized, single-arm, phase II clinical trial using Simon’s minimax two-stage design. The combination of anlotinib plus sintilimab is expected to result in an improved ORR of 35%. The null hypothesis, which stated that the true ORR was 15%, would be tested against a one-sided alternative of 35%, with 80% power and a type I error rate of 5%. In the first stage, the study accrued 15 patients. If less than two response or no response was observed, the study was terminated and deemed negative. Otherwise, an additional 13 patients would be accrued for the second stage. The study was considered positive if there were more than 9 responders among the 28 patients.

- 1. **Single-arm**

This trial design is a single-arm study without control group.

- 1. **Participants**

According to the inclusion criteria, all patients required to be treatment-naïve advanced CRCs

**3.3.1 inclusion criteria**

- Patients have histologically or cytologically confirmed advanced or recurrent CRC;
- No prior systematic anti-cancer treatment and relapse or metastases was occurred more than 12 months after adjuvant chemotherapy;
- Patients have measurable disease as defined by RECIST 1.1 as determined by investigator;
- Patient with a history of radiotherapy at least 3 months before on the day of providing consent, but the measurable lesion should not be within the scope of radiotherapy;
- Patients with age of 18-75yr;
- Patients with a performance status of 0,1or 2 on the Eastern Cooperative Oncology Group.；
- Patients with Life expectancy of more than 12 weeks;
- Patients must have the ability to understand and sign the written informed consent voluntarily;
- Female of childbearing potential who are negative in a pregnancy test within 7 days before enrollment. Both male and female patients should agree to use an adequate method of contraception (total abstinence, an intrauterine device or hormone releasing system, a contraceptive implant and an oral contraceptive) starting with the first dose of study therapy through 120 days after the last dose of study therapy. Duration will be determined when the subject is assigned to treatment.

**Exclusion criteria**

- Patients with dMMR/MSI-H;
- Patients with major surgery or severe trauma within 4 weeks before the first medication;
- Patients with hypersensitivity to the components in the study protocol;；
- Patients who are ready to give birth or are pregnant;
- Patients with brain metastases who are unable to accurately describe the condition;
- Patients received immune-suppressive drugs 2 weeks before initial treatment (inhaled cortisol or other steroid hormones≤10 mg/day prednisone or equivalent pharmacophysiologic doses were excluded);
- Planned live attenuated vaccine within 4 weeks prior to or during study treatment;
- Patients have received anlotinib or anti-PD-1 monoclonal antibody therapy or other therapies that act on T-cell co-stimulation targets or checkpoints;
- Within 6 months prior to the start of study treatment, the following diseases appeared: myocardial infarction, severe/unstable angina, NYHA grade 2 or above congestive heart failure, poorly controlled arrhythmias, etc;
- Active hepatitis;
- Bone marrow, liver and kidney function did not meet the requirements of chemotherapy as follows:

- Neutrophil count<1,500/mm3;

- Platelet count <80,000/mm3;

- Total bilirubin >1.5-times the upper limit of normal;

-ALT/AST>2.5-times the upper limit of normal for patients without liver

metastases; (5.0-times the upper limit of normal for patients with

liver metastases)

- Creatinine >1.5-times the upper limit of normal;

- Patients with cancers other than advanced colorectal cancer within five years prior to the start of treatment in this study. Cervical carcinoma in situ, cured basal cell carcinoma and bladder epithelial tumor were excluded;
- History of substance abuse, drug use, alcohol dependence;
- Patients without legal capacity or limited civil capacity;
- Patients with autoimmune diseases or organ transplantation;
- Other situations that the investigator deemed inappropriate for enrollment;
  1. **Study Time**

According to about 1 patient per month, it takes about 20 months. The total study period is about 2-3 year.

- 1. **Withdrawn from the study**

Withdrawal cases refer to patients who stop taking drugs for various reasons in this trial. Patients with the following conditions will withdraw from the study:

• Patient or his legal representative requests to withdraw;

• According to the opinion of the researcher, it is harmful to the health of patients to continue to participate in the study;

• Patients with severe poor compliance or serious violation of the protocol;

• Pregnancy should be reported as a serious adverse event;

• Other diseases occurred in the course of the study. According to the judgment of the investigators, the disease will obviously affect the evaluation of the clinical situation of patients, and it is necessary to stop the treatment of this scheme;

• Patients develop a new malignant tumors that need treatment;

• Patients lost to follow-up;

• The use of prohibited drugs or other drugs that may lead to toxic reactions or bias the research results according to the judgment of researchers;

• The study drug treatment was suspended for more than 21 days;

• The patient died.

All patients who withdraw from the study should record the reason for withdrawal in the case report form and the patient's medical record.

All patients who withdraw due to adverse events or laboratory abnormalities should be followed up until the adverse events recover or stabilize, and the subsequent results of the events should be recorded. If any patient died during the trial or within 30 days after completion of the trial, the investigator should be informed. The cause of death must be recorded in detail on the serious adverse event (SAE) report form within 24 hours.

- 1. **Procedures**

**3.6.1 The use of Medication**

Eligible patients were given sintilimab intravenously at a dose of 200mg on day 1, and anlotinib orally at a dosage of 12mg daily from days 1-14 every 3 weeks. Treatment was maintained until progressive disease (PD), treatment intolerance, death, development of intolerable toxicity, or withdrawal of consent, with one cycle as 3 weeks.

**3.6.2 Dose delay and dose adjustment of medication**

The toxicity of patients should be carefully monitored, and the dose should be adjusted according to the severity of adverse effect. It is suggested that the dose should be adjusted according to the most serious toxic reactions and laboratory abnormalities in the previous course of treatment or this course of treatment. The level of toxicity should be in accordance with NCI-CTCAE 5.0. If there are multiple toxic reactions, the dose should be adjusted according to the most serious toxic reaction.

The longest time allowed for treatment delay is 3 weeks, during which the symptoms should be relieved to CTCAE grade 1 or below. For patients with CTCAE grade 2 or more severe toxicity at baseline, symptoms should be reduced to baseline level.

Medication can be reduced / discontinued due to adverse events at any stage of the study.

If patients need to suspend treatment for more than 3 weeks due to treatment-related adverse reactions/toxicity, and the symptoms cannot be controlled even after the best supportive treatment, they should withdraw from the study.

If patients need to be hospitalized due to treatment-related adverse reactions / toxicities during the chemotherapy phase, all study drugs should be discontinued before all symptoms recover.

All adverse events should be graded according to NCI-CTCAE version 5.0.

If patients need to reduce anlotinib more than two times due to toxicity, they should withdraw from the study.

**3.6.2.1 dose adjustment of medication**

The management of toxicity involved supportive care, as well as prescheduled reduction in the dose of anlotinib, and interruptions of anlotnib dose until adverse events were tolerable (grade 2 or lower). Anlotinib could be reduced if patients had intolerable grade 2 or grade 3 treatment-related adverse events, which were judged by the investigators. The first dose-reduction of anlotinib was 10mg daily, the second dose-reduction of anlotinib was 8mg daily. If patients still could not tolerate to 8mg daily, anlotinib would be terminated. If anlotinib dose was reduced or interrupted, the dose could not be increases during subsequent cycles. Modifications to the sintilimab dose were not permitted. Patients who had intolerable adverse events (AEs) leading to the delay or cessation of one medication continued treatment with the other.

**3.6.2.2 The necessary laboratory test results at the beginning of the next cycle of chemotherapy,**

- - - ANC count ≥1.5×109/L,
    - Platelet count≥80×10^9^/L,
    - Serum creatinine <1.5×ULN,
    - Serum total bilirubin≤1.5-times the upper limit of normal for patients without liver metastases; (3.0-times the upper limit of normal for patients without liver metastases)；,
    - AST/ALT ≤2.5-times the upper limit of normal for patients without liver metastases; (5.0-times the upper limit of normal for patients without liver metastases)

**3.6.2.3 Dose adjustment** **plan for next cycle**

| Table 1: Dose adjustment for treatment-related toxicity | |
| --- | --- |
|  | Grade 3-4 adverse events |
| Anlotinib | The drug was stopped until the toxicity was reduced to Grade 2, and then the dose was reduced to 10mg qd at first dose-reduction, further 8mg qd at second dose-reduction. If not tolerate to 8mg qd, then anlotinib was terminated. |
| Sintilimab | Modifications to the sintilimab dose were not permitted. Sintilimab could be stopped or re-challenged according to the instruction of immune-AE. |
| If the patient cannot recover after 21 days delay, the treatment should be withdrawn. According to NCI-CTCAE 5.0 | |

**3.6.3 Concomitant treatment**

**3.6.3.1** **Unallowed Treatment**

• Except for Anlotinib plus Sintilimab, no other anticancer drugs are allowed,

• Research drugs (e.g. research antibiotics, antiemetics, etc.).

• In the course of treatment, any Chinese herbal medicine treatment with anti-tumor as the indication.

• Radiotherapy, except palliative radiotherapy for existing bone metastases.

**3.6.3.2 Allowed Treatment**

• Unconventional treatment (such as non-anticancer Chinese herbal medicine treatment or acupuncture), vitamins/trace elements can be used without affecting the observation of the endpoint of the study, which is controlled by the researcher.

• Patients can receive palliative treatment and supportive treatment for the original disease, such as diphosphate treatment.

• In order to relieve symptoms, radiotherapy was performed on the existing bone lesions (bone metastases existing at baseline).

• Patients with known bone metastases at baseline and need radiotherapy should complete radiotherapy before the first use of the study drug.

• Patients who had bone metastases at baseline, but did not need radiotherapy before the first use of the study drug, and then needed radiotherapy during the study were allowed to receive radiotherapy.

• Patients with bone metastases received radiotherapy at baseline, and they can receive radiotherapy when they need radiotherapy in the subsequent study.

• For patients undergoing radiotherapy, irinotecan should be suspended until radiotherapy is completed and the toxicity related to radiotherapy has improved.

**3.6.4 Treatment compliance**

For each subject, the dosage and administration time of each drug used in each course of treatment should be recorded in the CRF table. The reasons for delayed administration, drug reduction or missed administration should also be recorded in CRF.

Subjects' compliance with the treatment and protocol included voluntary compliance with all aspects of the protocol, including all blood collection required to comply with the safety assessment. If the subjects do not complete the follow-up or take medicine on time, they can be excluded from the study according to the opinions of the main researcher or the sponsor.

**3.7 Study Endpoint**

To assess the safety and activity of Anlotinib plus Sintilimab among treatment-naïve mCRC patients. Study endpoint included ORR, DCR, PFS, OS, safety.

**3.7.1 Efficacy Evaluation**

The antitumor effect will be evaluated in accordance with RECIST version 1.1 every 6 weeks.

The primary study endpoints for the study were objective response rate (ORR, complete or partial response according to RECIST version 1.1).

Secondary study endpoints included disease control rate (DCR, the proportion of patients who achieved a complete response, a partial response, or stable disease), progression-free survival (PFS), overall survival (OS) as well as safety.

PFS was defined as the time from therapy initiation to progression according to RECIST.

OS is defined as the time from therapy initiation to death from any cause.

Depth of response (DPR) was defined the percentage of maximum tumor reduction achieved in treatment compared with baseline. In case of CR, DPR is 100%.

Remission maintenance time was calculated in all PR or CR patients who were confirmed to meet RECIST 1.1 criteria. The duration of remission is the time from the first record to PR or Cr until death or disease progression (based on the first occurrence). As of the date of analysis, the deadline was the date of their last imaging evaluation.

**3.7.2 Safety**

All patients who have received at least one study medication will be regarded as the effective population for safety analysis. The physical examination results, vital signs, adverse events and laboratory abnormal values of patients will be summarized. All adverse events should be reported and graded according to NCI common adverse event Terminology Standard (CTCAE) version 5.0.

**3.7.3 Starting Time of study**

The time of starting the study was defined as the date of signing the informed consent.

**3.7.4.1 Treatment period**

- The screening phase should be conducted within 28 days before the start of study drug treatment.
- Treatment period refers to the interval from the use of the first drug treatment to the withdrawal of the patient from the study drug treatment.
- Patients will enter the post-treatment follow-up period after discontinuing the study drug treatment. This period should include contacting the patient every 3 months until 1 year after progression or recording the patient's death to collect the data of overall survival.

**3.7.4.2 Imaging Evaluation**

- Screening Phase: CT or MRI scans of the chest and abdomen must be performed within 28 days before enrollment, and the measurement of the tumor should be recorded in CRF according to RECIST 1.1
- Treatment Phase：CT or MRI scan should be performed at least every 6 weeks + / - 5 days to evaluate the lesions, and the measurement of tumor should be recorded in CRF according to RECIST 1.1. Other examinations were performed according to the symptoms.
- At the end of the study：CT or MRI scan was used to evaluate the lesion, and the measurement of tumor was recorded in CRF according to RECIST 1.1. Other examinations were performed according to the symptoms。

**3.8** **Trial Phase**

**3.8.1 Screening Phase**

Within 28 days before the start of study drug use

- - - Sign the informed consent before any operation related to the study;
    - The 12-lead electrocardiogram (ECG) in the baseline period was examined and signed by the researcher;
    - Only when there are neurological symptoms (suggesting brain metastasis), brain CT scan or brain MRI should be used to exclude brain metastasis;
    - Baseline tumor assessment: at least CT and MRI scans of the target lesions can be performed within 28 days before the start of study drug use

Screening period - within 7 days before the start of study drug treatment

- - - The stage and grade of the tumor at the time of diagnosis were recorded/confirmed;
    - Demographic information, medical history, surgical history, including previous anti-cancer treatment.
    - Complete physical examination (PE), including ECOG score, height, weight and detailed systematic examination.
    - Vital signs (including heart rate, blood pressure, respiratory frequency and body temperature).
    - All concomitant diseases, concomitant drugs and their indications were recorded
    - Blood routine examination: hemoglobin, platelet count, white blood cell count and classification, including the absolute number of neutrophils and lymphocytes.
    - Complete blood biochemical examination: including blood glucose, calcium, phosphorus, sodium, potassium, chlorine, creatinine, blood urea nitrogen (BUN), total protein, albumin, alanine aminotransferase (ALT), aspartate aminotransferase (AST), alkaline phosphatase, total bilirubin, etc.
    - Urine pregnancy test was performed on all women of childbearing age. Women who have been postmenopausal for at least one year and who have undergone sterilization are not required to undergo pregnancy tests.
    - Urine routine examination: including urine white blood cells, urine red blood cells, urine sugar, urine protein and occult blood

**3.8.2 Treatment Phase**

The treatment period was 21 days. The efficacy was evaluated every 2 courses. The laboratory examination, imaging assessment and AE assessment in the treatment stage are shown in the flow chart.。

**3.8.3** **At the end of the study**

At the end of study (not due to progression), the patients should be examined every 3 months until the disease progresses (see the flow chart).

**3.8.4 Temporary visit**

Temporary visit should be conducted according to clinical needs. The corresponding clinical laboratory abnormalities and adverse events should be recorded in CRF and original data. If multiple laboratory tests are performed on the same day, only one set of test values needs to be recorded in CRF. However, all abnormal values in repeated laboratory tests should be recorded in CRF.

**3.8.5** **Follow up after disease progression**

Get in touch with the patients every 3 months (visit or telephone) to get the information about the overall survival and chemotherapy after the study. Follow up should be carried out every 3 months until one year after the patient progressed or died. The following information should be obtained during each follow-up:

• The survival status of the patients.

• Disease status and progression date (if applicable).

• Date of death (if applicable).

• All new anticancer treatments were recorded.

Note: CRCs Patients with prognosis disease should be treated like other patients with advanced colorectal cancer.

**3.9 Data quality**

In order to follow the guidelines of clinical practice (GCP), investigator will take responsibility of ensure compliance with the study protocol, GCP and relevant laws including on-site inspection of the completeness and clarity of the case report form (CRF), cross checking with the original documents, and clarification of management matters.。

**3.10 Data File**

The data in the case report form (CRF) must be consistent with the original documents, or be directly recorded in the CRF. In the case of being directly recorded in the CRF, the recorded content will be regarded as the original data. The parameters of the original data must be verified, and the information of the data source must be recorded. The research documents and all the original materials should be kept.

1. **Ethical and legal issues**

**4.1 Ethics Committee (EC) or institutional review board (IRB)**

According to the requirements of GCP, Chinese laws and regulations and relevant organizations, the study should obtain approval documents from the corresponding ethics committee/institutional review committee before the start of the research. If necessary, the extension, amendment or review of the ethics committee's approval must be obtained and handed over to the researcher. The study protocol and all amendments were approved by the Research Ethics Board of Shanghai Changzheng Hospital and was done in accordance with the Declaration of Helsinki. All patients were required to provide written informed consent.

**4.2 The ethical guidance of this study**

The procedures for operation, evaluation and documentation involved in this study protocol are to ensure that researchers follow the guidelines of clinical practice and the guidelines detailed in the Helsinki declaration. The implementation of this study will also follow the corresponding laws and regulations in China.

The investigators were not allowed to modify the study protocol without consent. However, in case of emergency, in order to remove the risk factors for subjects, investigators can deviate from or change the study protocol without the consent or support of the ethics committee/institutional review committee/sponsor. The deviation or change and its reasons should be submitted to the ethics committee/institutional review committee/sponsor as soon as possible, and if appropriate, the proposal modification proposal. The investigators must explain all deviations or changes in the study protocol.

**4.3 Subject information and informed consent**

The subjects should be provided with the main information of the study and informed consent. Prior to the start of the study, the investigators must provide the subjects with the written approval of the ethics committee/institutional review committee/approval of the informed consent and all other written information. The ethics committee/institutional review committee approval and the approved subject instructions/informed consent must be filed in the study document together.

Informed consent must be obtained before implementing any specific study steps. The date when the subjects participated in the study and signed the informed consent form should be recorded in the corresponding documents of the subjects.

**4.4 Secrecy**

All records of the patient's identity are kept confidential and will not be made public to the extent permitted by relevant laws and/or regulations.

The name of the subject will not be provided. Only subject number and abbreviation were recorded in case report form. If the subject's name appears in any other document (e.g. pathology report), the copy of the document must be erased. Research reports stored in computers must comply with local data protection laws. When the results of the study are published, the identity of the subjects will also be kept confidential.

The investigator will keep a list to identify the subjects' records.

1. **Statistical methods**

**5.1 Statistics analysis plan**

**5.1.1 The population for analysis**

Efficacy analysis will be conducted in the intention to treat population (ITT) and efficacy-evaluable population. The patients who did not have at least one post-baseline efficacy assessment were defined as not assessable for efficacy and were ruled out from the efficacy-evaluable population. The safety analysis included all patients in ITT population who had received at least one study medication. The analysis population of ORR refers to all patients with partial remission or complete remission determined by RECIST 1.1 in ITT population and efficacy-evaluable population.

**5.1.2 Baseline and demographic characteristics**

The baseline, demographic characteristics, baseline tumor characteristics, medical history, previous anticancer treatment, combination therapy, vital signs and termination of the trial were summarized. For continuity assessment, the mean, standard deviation, distribution range and median will be calculated. Absolute value, frequency and percentage will also be calculated.

**5.1.3 Efficacy analysis**

Efficacy analysis will be conducted in the intention to treat population (ITT) and efficacy-evaluable population.

**5.1.3.1 Primary Study Endpoint**

Primary endpoint：Objective Response Rate (ORR, complete or partial response according to RECIST version 1.1).

**5.1.3.2** **Secondary study endpoints**

Secondary study endpoints included disease control rate (DCR, the proportion of patients who achieved a complete response, a partial response, or stable disease), progression-free survival (PFS), overall survival (OS) as well as safety.

PFS was defined as the time from therapy initiation to progression according to RECIST.

OS is defined as the time from therapy initiation to death from any cause.

Depth of response (DPR) was defined the percentage of maximum tumor reduction achieved in treatment compared with baseline. In case of CR, DPR is 100%.

Remission maintenance time was calculated in all PR or CR patients who were confirmed to meet RECIST 1.1 criteria. The duration of remission is the time from the first record to PR or Cr until death or disease progression (based on the first occurrence). As of the date of analysis, the deadline was the date of their last imaging evaluation.。

Kaplan Meier method was used to get the expected survival curve of the patients including PFS and OS.

**5.1.3.3** **Exploration biomarker analysis**

PD-L1 expression, TMB, molecular alteration profile, tumor immune-microenvironment, plasma exosome miRNA.

**5.1.3.4 Safety analysis**

The adverse events will be summarized according to the NCI-CTCAE 5.0. The adverse events will be summarized according to the severity of the adverse event associated with study drug. The descriptive summary of laboratory values is mainly for abnormal values. Laboratory abnormalities will also be summarized according to the most severe grade in NCI-CTCAE 5.0.

6 **Adverse Events**

**6.1** **Detection of adverse events**

The adverse events of the subjects must be closely monitored including clinical symptoms and laboratory tests. The severity grade and relationship with the drugs should be evaluated. The investigator was responsible for evaluating the relationship between all adverse events and the drugs. However, the primary investigator can entrust other researchers participating in this study to judge, but still need to be responsible for this. The researcher must provide a list of qualified and entrusted personnel.。

**6.2 Definition of adverse events**

**6.2.1 adverse events**

An adverse event is any adverse medical event that occurs to a patient or a subject when treated with the study drug. There may be no causal relationship between adverse events and treatment. Therefore, an adverse event can be any adverse and meaningless sign (including abnormal laboratory test), symptom or disease temporarily associated with the study drug used, regardless of whether the event is drug-related or not.

Adverse events (whether drug-related or not) in human body may include the following aspects:

• Adverse events occurred during the use of drugs by professionals;

• Adverse events caused by overdose (whether intentional or unintentional);

• Adverse events caused by drug abuse;

• Adverse events caused by discontinuation of medication;

• Adverse events that may be purely due to the patient's participation in the study (e.g. adverse events due to withdrawal of antihypertensive drugs during washout period or serious adverse events), which must be reported as adverse events even if they are not related to the study medication.

The adverse events were described according to CTCAE 5.0.

**6.2.2 Serious adverse events**

Serious adverse events refer to all adverse events that meet one of the following conditions：

• Causing death;

• Life threatening;

• Causes the patients to be hospitalized or the hospitalized patient prolongs the hospitalization time;

• Cause permanent or obvious loss of working ability or disability;

• Congenital malformation or defect;

• Serious medical events

Life threatening**:** The term "life-threatening" is defined as "serious" and refers to the risk of death of the subject when an adverse event occurs. It does not refer to those adverse events that may lead to death if the situation is assumed to worsen.

Hospitalization: all adverse events causing hospitalization or prolonging the hospitalization time were defined as “serious adverse event”, expect for the following aspects:

- - - Stay in hospital for no more than 12 hours
      or
    - Hospitalization was planned before the start of this study (i.e. scheduled surgery or elective surgery before the start of this study);

or

- - - Hospitalization was not associated with adverse events (e.g., hospitalization for convalescent purposes).

**Disability：**someone's ability to engage in daily life is seriously impaired.

**Serious medical events**：Any adverse event may harm the subject and may require intervention to prevent a more serious situation, which can be considered as serious medical event. Serious medical events should be determined according to the WHO terminology of adverse events. The reason why these events are reported as serious adverse events is that they may be related to the serious disease stats, and as SAE reports, it can ensure special attention and promote necessary actions compared with other reporting methods.

**6.2.3 Unexpected adverse events**

Unexpected adverse events refer to any adverse events which are inconsistent with the researcher's Manual (or the instructions in the package of the marketed products). It is also an integral part of the report of unexpected adverse events to supplement the important information about the characteristics or severity of known and recorded adverse events. For example, events that are more special or serious than those described in the researcher's manual should be considered "unexpected". For example: (a) interstitial nephritis followed by acute renal failure of records adverse events; And (b) hepatitis first reported as acute hepatic necrosis.

**6.2.4 Relationship between adverse events and research drugs**

The evaluation of the relationship between an adverse event and the study drug is a comprehensive clinical judgment based on all the information obtained when completing the case report form.

Evaluation of "irrelevant" situations may include:

- - 1. There are clear other explanations, such as traumatic bleeding at the surgical site;
       or
    2. Unreasonable, for example, the subject was hit by a car, but there was no evidence that the drug-induced disorientation caused the event; Or cancer developed just a few days after the start of administration.

A "yes" assessment indicates there is a reasonable reason that the adverse event may be related to the study medication.。

When the investigators evaluated the relationship between adverse events and study drugs, the following factors should be considered：

– The adverse event should occur after administration. The interval between medication and the occurrence of the adverse event should be considered in clinical evaluation.

– The event disappeared after stopping administration (stopping stimulation) and recurred after re-administration (repeated stimulation): when analyzing the clinical process of the suspicious event, the response of the subject after stopping administration (stopping stimulation) or after re-Administration (repeated stimulation) should be fully considered.

– Basic diseases, concomitant diseases and intermittent diseases: the natural course of disease, treatment process and all other diseases that patients may have in each report should be evaluated;

– Combined medication or treatment: other drugs taken by the subject or other treatments received by the subject should be checked to determine whether one of them may cause the adverse event;

– Known response patterns of a class of drugs: clinical/preclinical

–Pharmacology and pharmacokinetics of the test drug: the pharmacokinetic characteristics (absorption, distribution, metabolism, and excretion) of the test drug should be considered in combination with the individual pharmacodynamic response of each subject.

**6.2.5 Record of adverse events**

All adverse events occurred after the informed consent should be signed by the subjects and must be completely recorded in the case report form each subject.

The records must be supported by original data. Laboratory abnormalities associated with clinically treatment (such as those that lead to early withdrawal from the study, need treatment or cause obvious clinical manifestations, or those are considered to be associated with treatment by investigators) should be reported as adverse events. Each event should be described in detail, including the start and end time, severity, relationship with the treatment, measures and the outcome of the event.

**6.3 Report of serious adverse events**

Serious adverse events (SAEs) meeting the definition, including laboratory abnormalities meeting the definition of serious adverse events, occurring within 30 days from the signing of the informed consent form to the completion of the last medication, must be reported to the designated person in the study document immediately (within 24 hours after the researchers know). The serious adverse event report form must also be completed and sent to the person specified in the study document within 24 hours of the investigator's knowledge.

Each serious adverse event should be followed up until it is resolved or stabilized, and an updated report should be submitted to the designated person. A single grade 4 laboratory abnormality (according to CTCAE version 4.0) is not reported as a serious adverse reaction unless the investigator considers that the abnormality has met the criteria of the international coordination meeting for serious adverse events (see definition in section 6.3.2). The level 4 laboratory examination abnormalities of CTCAE version 4.0 as the manifestation of the disease in the baseline period should not be reported as serious adverse events, especially when these abnormalities are still allowed or not excluded by the protocol. If there is doubt as to whether the abnormality should be reported as a serious adverse event, the researcher may consult the research supervisor. The abnormal laboratory examination of CTCAE level 4 should be recorded in the "laboratory data" page and checked regularly by the medical inspector.

According to local laws and regulations, serious adverse events must be reported to the ethics committee and FDA.

**7 Appendix**

**7.1 ECOG**

| **Score** | **Description** |
| --- | --- |
| 0 | Fully active, able to carry on all pre-disease performance without restriction (Karnofsky 90-100) |
| 1 | Restricted in physically strenuous activity but ambulatory and able to carry out work of a light or sedentary nature, e.g., light house work, office work (Karnofsky 70-80) |
| 2 | Ambulatory and capable of all selfcare but unable to carry out any work activities. Up and about more than 50% of waking hours (Karnofsky 50-60) |
| 3 | Capable of only limited selfcare, confined to bed or chair more than 50% of waking hours (Karnofsky 30-40) |
| 4 | Completely disabled. Cannot carry on any selfcare. Totally confined to bed or chair (Karnofsky 10-20) |

**7.2 TNM stage**

The tumor stage of each patient was determined based on the AJCC-TNM system.

1. Definitions for T, N, M

***Primary Tumor（T）***

- - - TX：Primary tumor cannot be assessed
    - T0：No evidence of primary tumor
    - Tis：carcinoma in situ: intramucosal carcinoma (involvement of lamina propria with no extension through muscularis mucosae)
    - T1：Tumor invades the submucosa (through the muscularis mucosa but not into the muscularis propria)
    - T2：Tumor invades the muscularis propria
    - T3：Tumor invades through the muscularis propria into the pericolorectal tissues
    - T4：Tumor invades the visceral peritoneum or invades or adheres to adjacent organ or structure
    - T4a：Tumor invades through the visceral peritoneum (including gross perforation of the bowel through tumor and continuous invasion of tumor through areas of inflammation to the surface of the visceral peritoneum)
    - T4b：Tumor directly invades or is adheres to adjacent organs or structures.

***Regional Lymph Nodes（N）***

- - - NX：Regional lymph nodes cannot be assessed
    - N0：No regional lymph node metastasis
    - N1：one to three regional lymph node are positive
    - N1a：one regional lymph node is positive
    - N1b：two or three regional lymph nodes are positive
    - N1c：No regional lymph node are positive, but there are tumor deposits in the subserosa, mesentery, or nonperitonealized pericolic or perirectal/mesorectal tissues
    - N2：Four or more regional lymph nodes are postive
    - N2a：Four to six regional lymph nodes are positive
    - N2b：Seven or more regional lymph nodes are positive

***Distant Metastasis（M）***

- - - MX：Distant metastasis cannot be assessed
    - M0：No distant metastasis by imaging, etc.; no evidence of tumor in distant sites or organs
    - M1：metastasis to one or more distant sites or organs or peritoneal metastasis is identified.
    - M1a：Metastasis to one site or organ is identified without peritoneal metastasis
    - M1b：Metastasis to two or more sites or organs is identified without peritoneal metastasis
    - M1c：Metastasis to the peritoneal surface is identified alone or with other site or organ metastases

**Anatomic stage/prognostic Groups**

***Stage 0*** Tis, N0, M0

***Stage IA*** T1, N0, M0

***Stage IB*** T2, N0, M0

***Stage IIA*** T3, N0, M0

***Stage IIB*** T4a, N0, M0

***Stage IIC*** T4b, N0, M0

***Stage IIIA*** T1-2, N1/N1c, M0

T1, N2a, M0

***Stage IIIB*** T3-4a, N1, M0

T2-3, N2a, M0

T1-2, N2b, M0

***Stage IIIC*** T4a, N2a, M0

T3-4a, N2b, M0

T4b, N1-2, M0

***Stage IVA*** any T, any N, M1a

***Stage IVB*** any T, any N, M1b

**7.3 RECIST criteria**

**Measurable Lesion：**Tumour lesions: Must be accurately measured in at least one dimension (longest diameter in the plane of measurement is to be recorded) with a minimum size of:

• 10 mm by CT scan (CT scan slice thickness no greater than 5 mm; see Appendix II on imaging guidance).

• 10 mm caliper measurement by clinical exam (lesions which cannot be accurately measured with calipers should be recorded as non-measurable).

• 20 mm by chest X-ray.

Malignant lymph nodes: To be considered pathologically enlarged and measurable, a lymph node must be 15 mm in short axis when assessed by CT scan (CT scan slice thickness recommended to be no greater than 5 mm).

**Non-measurable Lesion：**All other lesions, including small lesions (longest diameter <10 mm or pathological lymph nodes with 10 to <15 mm short axis) as well as truly non-measurable lesions. Lesions considered truly non-measurable include: leptomeningeal disease, ascites, pleural or pericardial effusion, inflammatory breast disease, lymphangitic involvement of skin or lung, abdominal masses/abdominal organomegaly identified by physical exam that is not measurable by reproducible imaging techniques.

**Target Lesion：**At most 2 lesions can be selected as target lesions in all measurable lesions of each organ, and at most 5 representative lesions can be selected as target lesions in all involved organs. All target lesions should be recorded and measured at baseline. These five lesions should be selected according to their size (the longest diameter of the lesion) and the repeatability of accurate measurement (whether imaging technology or clinical measurement). The sum of the longest diameter (LD) of all target lesions will be taken as the total LD at baseline. The total LD in the baseline period will be used as the reference value to objectively evaluate the tumor response by measuring the size of future lesions. If there are more than 5 measurable lesions, those not selected as target lesions will be regarded as non target lesions together with unmeasurable lesions

**Non-target lesion：**It includes all unmeasurable lesions (or sites) and all measurable lesions except the five selected target lesions. These lesions need not be measured, but should be recorded as "present" or "not present" at baseline and at each follow-up.

**Evaluation of response**

Complete Response (CR): Disappearance of all target lesions. Any pathological lymph nodes (whether target or non-target) must have reduction in short axis to <10 mm.

Partial Response (PR): At least a 30% decrease in the sum of diameters of target lesions, taking as reference the baseline sum diameters.

Progressive Disease (PD): At least a 20% increase in the sum of diameters of target lesions, taking as reference the smallest sum on study (this includes the baseline sum if that is the smallest on study). In addition to the relative increase of 20%, the sum must also demonstrate an absolute increase of at least 5 mm. (Note: the appearance of one or more new lesions is also considered progression).

Stable Disease (SD): Neither sufficient shrinkage to qualify for PR nor sufficient increase to qualify for PD, taking as reference the smallest sum diameters while on study.**靶The evaluation of Target lesion and non-target lesion**

| **Target lesion** | **non-target lesion** | **New lesion** | **Response** | **requirements** |
| --- | --- | --- | --- | --- |
| CR | CR | No | CR | confirmed after more than 4 weeks |
| CR | Non-CR/non-PD | No | PR | confirmed after more than 4 weeks |
| PR | Non-PD | No | PR |  |
| SD | Non-PD | No | SD | At least 4 weeks from baseline |
| PD | Any | No or yes | PD | No-SD/PR/CR |
| Any | PD | No or yes | PD |  |
| Any | Any | Yes | PD |  |

**Remission time**

The remission time should be calculated from the time when CR or PR (no matter who was recorded first) was first met, until the date of recurrence or progression of the disease was first recorded.。

**Time of Stable disease**

The stable time of the disease should be calculated from the time of the beginning of treatment until the progression of the disease. The minimum measured value of the lesion from the beginning of treatment was used as the reference.

**7.5 Flow-chart of study**

|  | Before-treatment | 21days for one cycle | | | | At the end of study | At the end of study to PD | After PD_10_ |
| --- | --- | --- | --- | --- | --- | --- | --- | --- |
|  |  | 1^st^ cycle | 2^nd^  cycle | 3^rd^  Cycle | 4^th^  to PD or treatment terminated |  |  |  |
| Items | Screening | Day1 | Day1 | Day1 | Day1 | Within 7 days | Every 8 weeks | Every 3 months |
| Informed consent of patients | X（within 28 days） |  |  |  |  |  |  | The following information was collected by telephone or outpatient visit every 2 months  1. Survival information of patients;  2. Changes in the treatment of cancer, including all new treatments |
| Medical History _1_ | X（within 7 days） |  |  |  |  |  |  |  |
| Complete physical examination_2_ | X（within 7 days） |  |  |  |  |  |  |  |
| Inclusion/exclusion Criteria | X |  |  |  |  |  |  |  |
| Random | X |  |  |  |  |  |  |  |
| Vital signs and ECOG |  | X | X | X | X | X | X |  |
| ECG | X（within 28 days） |  | X | X | X | X |  |  |
| CT and MRI _3_ | X（within 28 days） |  | | | |  |  |  |
| Imaging Evaluation_4_ | X（within 28 days） | X （每6周)-- -------------🡪 | | | | | |  |
| Hematological Examination_5_ | X（within 7 days） |  | X | X | X | X |  |  |
| Biochemical examination_6_ | X（within 7 days） |  | X | X | X | X |  |  |
| Urine test_7_ | X（within 7 days） |  | X | X | X | X |  |  |
| Serum pregnancy test | X（within 7 days） |  | | | | |  |  |
| QLQ_8_ |  | X_9_ |  |  | Before 5^th^ cycle or 9^th^ cycles | X |  |  |
| Use Drug |  | X | X | X | X | X |  |  |
| AE evaluation | -- -------------🡪 | | | | | | |  |
| Concomitant disease and drug combination | -- -------------🡪 | | | | | | |  |

1. Complete medical history, demographic data, previous surgery history, concomitant diseases, allergic history, and smoking history.
2. Complete physical examination, ECOG score, NYHA heart disease classification, height, weight, vital signs and detailed examination of the body system
3. Only patients with neurological symptoms at baseline required head CT / MRI to exclude brain metastases
4. During chemotherapy, tumor assessment is performed every 8 weeks. After treatment, CT scan was required every 8 weeks until the disease progressed. If the patient withdraws from the study due to disease progression, a review of CT scan is not required at the end of the study。
5. Hematological test: hemoglobin, hematocrit, platelet, leukocyte, neutrophil absolute value, lymphocyte absolute value, which can be checked one day before each course of treatment。
6. Biochemical tests: blood glucose, calcium, phosphorus, sodium, potassium, chlorine, creatinine, blood urea nitrogen, total protein, albumin, alanine aminotransferase, aspartate aminotransferase, alkaline phosphatase, total bilirubin, etc., which can be checked one day before each course of treatment.
7. Urine tests: urine specific gravity, PH value, urine sugar, urine protein and occult blood. In the screening period, urine test is required in the laboratory, and urine test paper can be used in the subsequent course of treatment.
8. Patients should be followed up every 3 months until 1 year or death.

**8.Reference**

[1] Chen W, Zheng R, Baade PD, et al. Cancer statistics in China, 2015. CA Cancer J Clin. 2016. 66(2): 115-32.

[2] Van Cutsem E, Cervantes A, Nordlinger B, Arnold D, ESMO Guidelines Working Group. Metastatic colorectal cancer: ESMO Clinical Practice Guidelines for diagnosis, treatment and follow-up. Ann Oncol. 2014. 25 Suppl 3: iii1-9.

[3] Clinical Practice Guidelines in Oncology (NCCN Guidelines): Colon Cancer Version 2. 2019 .

[4] Wang FH, Shen L, Li J, et al. The Chinese Society of Clinical Oncology (CSCO): clinical guidelines for the diagnosis and treatment of gastric cancer. Cancer Commun (Lond). 2019. 39(1): 10.

[5] Guidelines for the Diagnosis and Treatment of Colorectal Cancer by the Chinese Society of Clinical Oncology. 2019

[6] Le DT, Uram JN, Wang H, et al. PD-1 Blockade in Tumors with Mismatch-Repair Deficiency. N Engl J Med. 2015. 372(26): 2509-20.

[7] Eng C, Kim TW, Bendell J, et al. Atezolizumab with or without cobimetinib versus regorafenib in previously treated metastatic colorectal cancer (IMblaze370): a multicentre, open-label, phase 3, randomised, controlled trial. Lancet Oncol. 2019. 20(6): 849-861.

[8] Grothey A, Van Cutsem E, Sobrero A, et al. Regorafenib monotherapy for previously treated metastatic colorectal cancer (CORRECT): an international, multicentre, randomised, placebo-controlled, phase 3 trial. Lancet. 2013. 381(9863): 303-12.

[9] Li J, Qin S, Xu R, et al. Regorafenib plus best supportive care versus placebo plus best supportive care in Asian patients with previously treated metastatic colorectal cancer (CONCUR): a randomised, double-blind, placebo-controlled, phase 3 trial. Lancet Oncol. 2015. 16(6): 619-29.

[10] 2017 ESMO Annual Meeting #1198P.
