# Supplementary material for: Anlotinib plus sintilimab as first-line treatment for patients with advanced colorectal cancer (APICAL-CRC): an open-label, single-arm, phase II trial
Source: Signal Transduct Target Ther. 2025 Sep 16;10:301. doi: 10.1038/s41392-025-02383-9 (PMC12436658; doi:10.1038/s41392-025-02383-9)
Supplement: Supplementary file 2 — Supplementary Materials [file 41392_2025_2383_MOESM2_ESM.docx]

Supplementary Materials for

Anlotinib plus sintilimab as first-line treatment for patients with advanced colorectal cancer (APICAL-CRC): an open-label, single-arm, phase II trial

Zhan Wang, M.D., Bao-Dong Qin, M.D., Chen-Yang Ye, M.D., Miao-Miao Wang, M.D., Ling-Yan Yuan, M.D., Hou-Shan Yao, M.D., Xiao-Dong Jiao, M.D., Ke Liu, M.D., Wen-Li Zhou, M.D., Wen-Xing Qin, M.D., Li Sun, M.D., Wei-Ping Dai, M.D., Yan Ling^1^, M.D., Ying Wu, M.D., Shi-Qi Chen, M.D., Ying-Fu Zhang, M.D., Dong-Min Shi, M.D., Xiao-Peng Duan, M.D., Xue Zhong, M.D., Xi He, M.D., Wen-Xin Zhai, M.D., Bei Zhang, M.D., Da-Dong Zhang, M.D., Ning Gao, M.D., Yuan-Sheng Zang, M.D.

Correspondence to: [doctorzangys@163.com](mailto:doctorzangys@163.com)

**Figure S1:** Clinical activity endpoints (ORR, PFS, OS) analysis stratified by ECOG, liver metastases. **a**: comparison analysis stratified by ECOG-PS (PS 0-1 versus PS 2). **b**: stratified by the presence or absence of liver metastases.

**Figure S2:** Oncoprint of concomitant mutations, PD-L1 expression, TMB, MATH in the treatment-naïve mCRC patients from the efficacy-evaluable cohort. The molecular alteration profile of 27 patients was assessed based on a customized next-generation sequencing panels targeting 733 cancer-related genes using FFPE tissues.

**Figure S3:** The association between tertiary lymphoid structure (TLS) and clinical response. No significant difference in TLS between responders (PR+CR) than non-responders (PD+SD). Tertiary lymphoid structure has been assessed in 26 out of 30 patients. All 26 patients have tumor response results, while 25 patients were used to assess the correlation between PFS and tertiary lymphoid structure due to one patient have not developed PFS event.

**Figure S4:** Baseline plasma exosomal miRNAs associated with response to study regimen, with KEGG pathways enriched with target genes of the six miRNAs (top 30).

**Table S1:** The baseline characteristics between patients with PS 0-1 and PS 2

**Table S2**: Multivariate Cox regression analysis for PFS and OS in this cohort.

**Table S3:** Inclusion criteria and exclusion criteria of this trial

**Table S4:** Comparison of immune cell infiltration in TIME with clinical response.

**Table S5:** Antibodies used in the study

**Method S1:** NGS detection for molecular alteration profile of each enrolled mCRC patients.

**Method S2:** Tumor microenvironment (TME) by multiplex immunofluorescence (mIF).

**Method S3:** Plasma exosomal miRNA detection

**Figure S1:** Clinical activity endpoints (ORR, PFS, OS) analysis stratified by ECOG, liver metastases. **a**: comparison analysis stratified by ECOG-PS (PS 0-1 versus PS 2). **b**: stratified by the presence or absence of liver metastases.


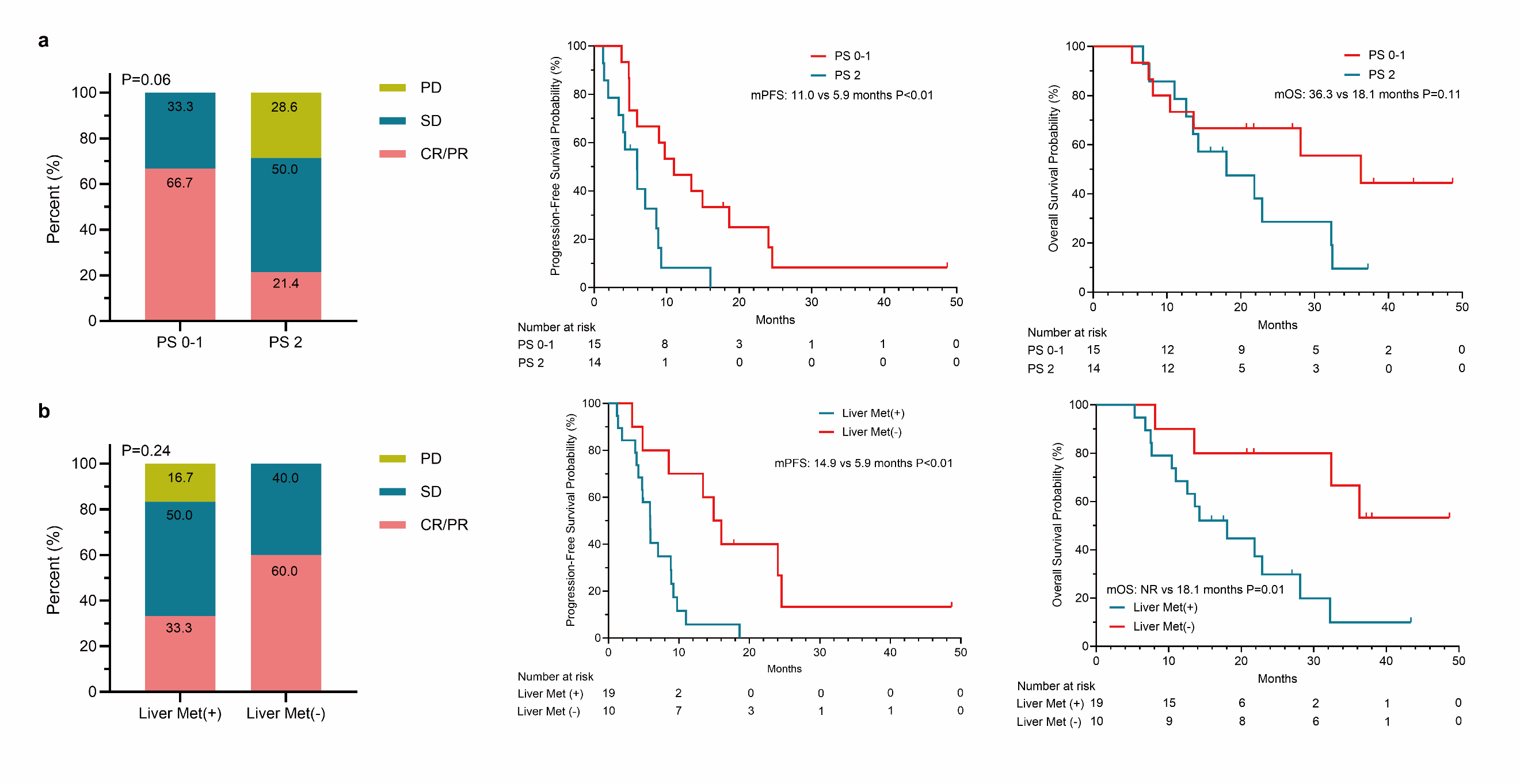


**Figure S2:** Oncoprint of concomitant mutations, PD-L1 expression, TMB, MATH in the treatment-naïve mCRC patients from the efficacy-evaluable cohort. The molecular alteration profile of 27 patients was assessed based on a customized next-generation sequencing panels targeting 733 cancer-related genes using FFPE tissues.

**
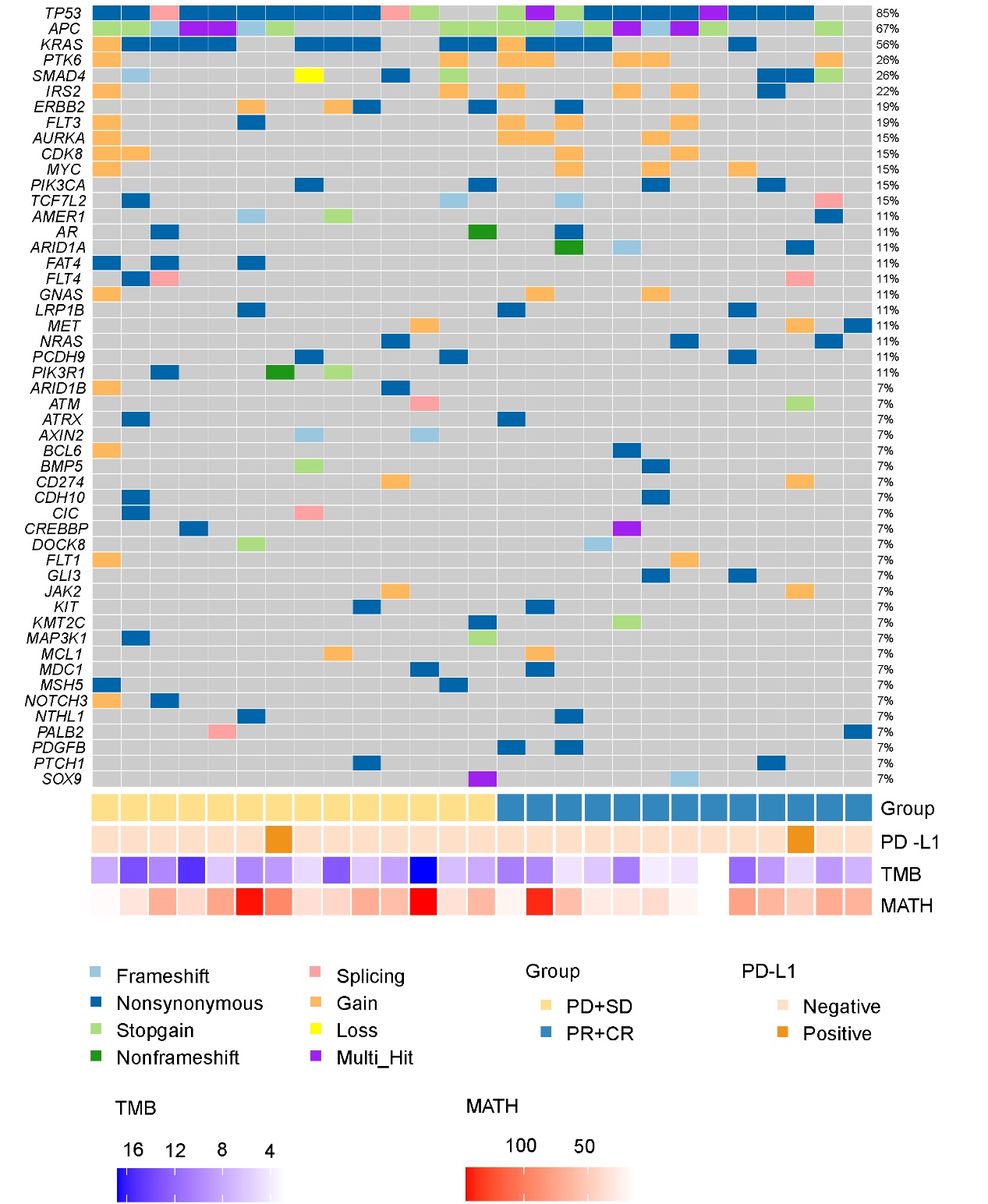
**

**Figure S3:** The association between tertiary lymphoid structure (TLS) and clinical response. No significant difference in TLS between responders (PR+CR) than non-responders (PD+SD). Tertiary lymphoid structure has been assessed in 26 out of 30 patients. All 26 patients have tumor response results, while 25 patients were used to assess the correlation between PFS and tertiary lymphoid structure due to one patient have not developed PFS event.


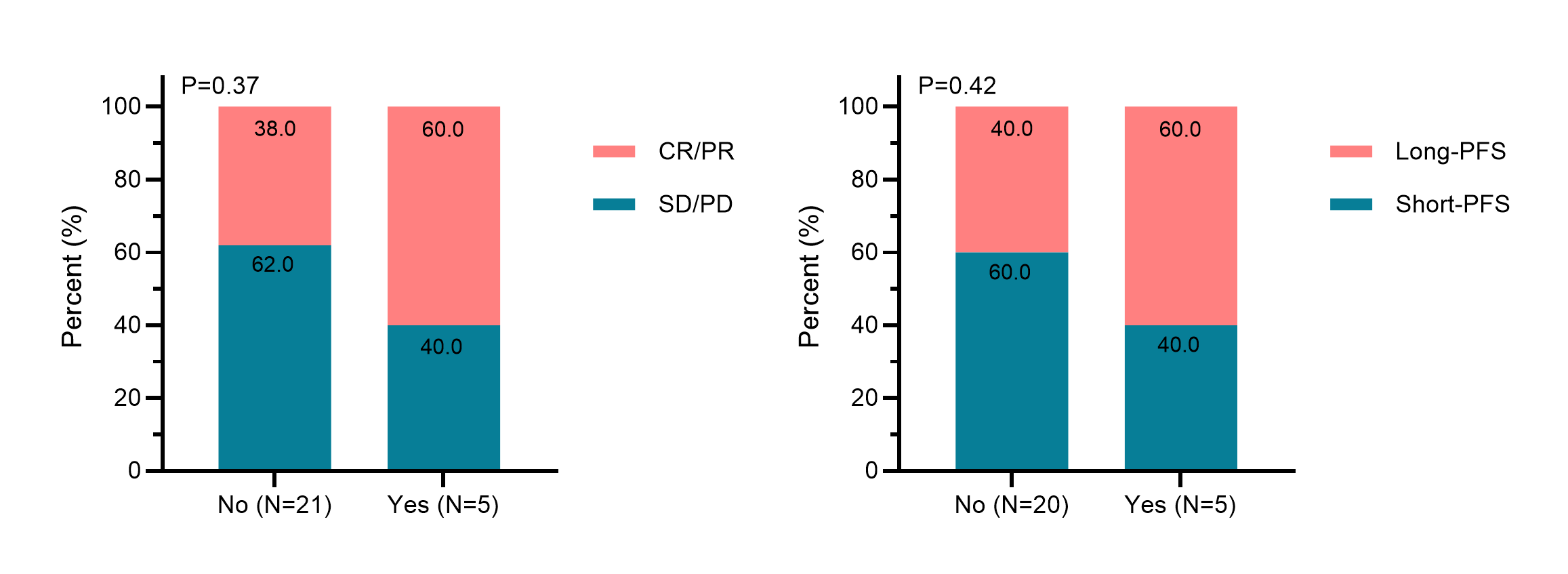


**Figure S4:** Baseline plasma exosomal miRNAs associated with response to study regimen, with KEGG pathways enriched with target genes of the six miRNAs (top 30).

**
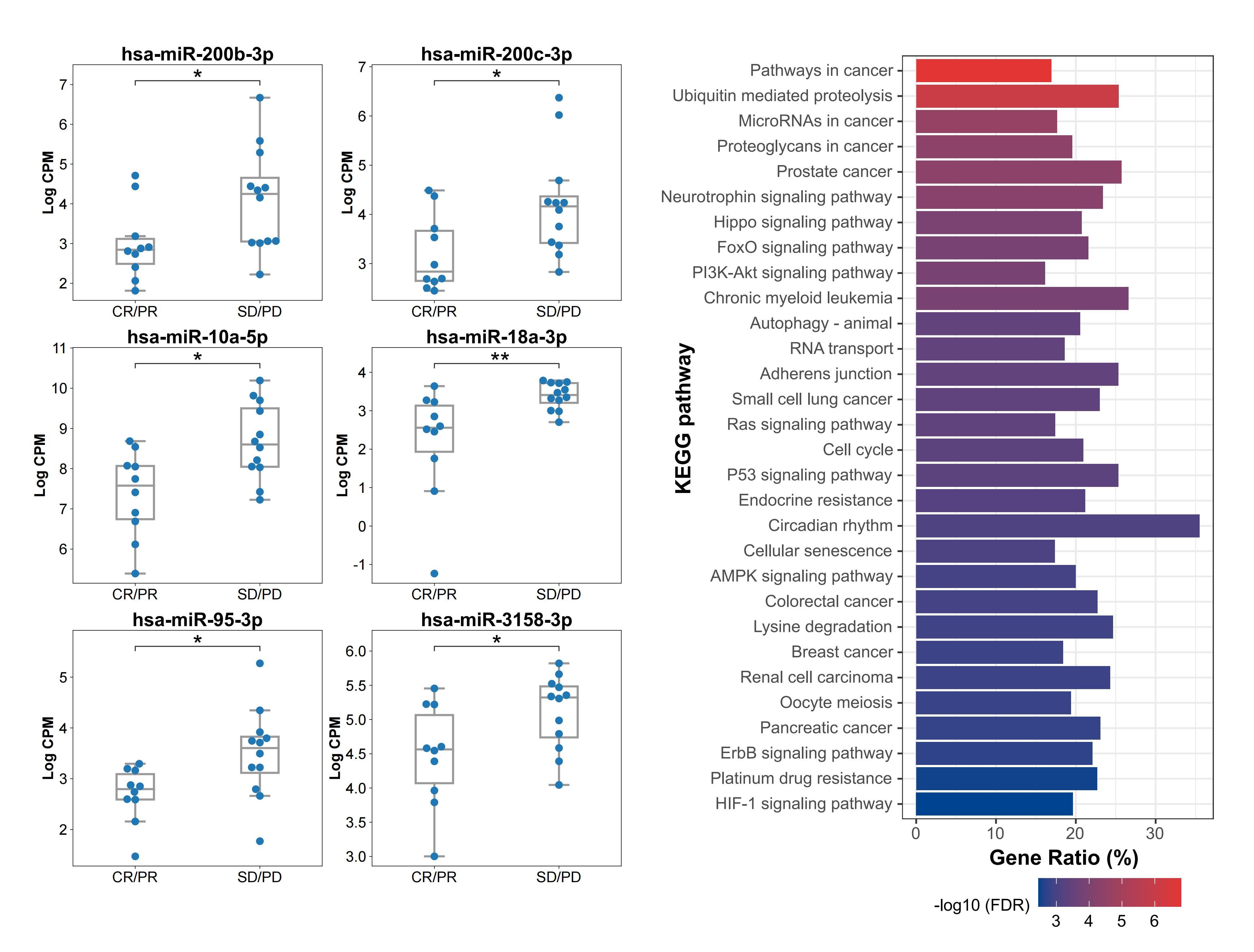
**

**Table S1**: The baseline characteristics between patients with PS 0-1 and PS 2

| **Characteristics** | **PS 0-1 (N=15)** | **PS 2 (N=15)** | **P value** |
| --- | --- | --- | --- |
| Age |  |  |  |
| Median (Range) | 57 (38-69) | 63 (53-75) | 0.04 |
| Sex |  |  |  |
| Female | 3 | 10 | 0.03 |
| Male | 12 | 5 |  |
| Comorbidity (e.g., diabetes, hypertension, etc) |  |  |  |
| No | 7 | 12 | 0.22 |
| 1 | 5 | 2 |  |
| ≥2 | 3 | 1 |  |
| Primary site |  |  |  |
| Left Colon | 4 | 2 | 0.51 |
| Right Colon | 4 | 6 |  |
| Rectum | 7 | 7 |  |
| Number of metastatic site (Range) |  |  |  |
| 1 | 10 | 7 | 0.46 |
| ≥2 | 5 | 8 |  |
| Metastatic site |  |  |  |
| Liver | 8 | 11 | 0.08 |
| Lung | 4 | 5 |  |
| Distant lymph node | 5 | 6 |  |
| Peritoneum | 1 | 4 |  |
| Other | 3 | 4 |  |
| Isolated Lung metastases | 1 | 2 | 0.99 |
| Number of lesions | 4 | 7 (6-8) | 0.33 |
| Maximum diameter (mm) | 14.6 | 61.0 (31.6-90.4) | 0.53 |
| Isolated Liver metastases | 7 | 4 | 0.45 |
| Number of lesions | 7 (1-50) | 2 (2-33) | 0.74 |
| <4 lesions | 2 | 2 | 1.00 |
| Maximum diameter (mm) | 55 (23-90.4) | 26.2 (24.6-114.2) | 0.87 |
| MMR status |  |  |  |
| dMMR | 0 | 0 | NA |
| pMMR | 15 | 15 |  |
| KRAS mutation |  |  |  |
| Yes | 5 | 10 | 0.14 |
| No | 10 | 5 |  |
| BRAF mutation |  |  |  |
| Yes | 1 | 0 | 1.00 |
| No | 14 | 15 |  |
| FLT4 mutation |  |  |  |
| Yes | 2 | 1 | 0.99 |
| No | 13 | 14 |  |
| PIK3R1 mutation |  |  |  |
| Yes | 1 | 2 | 0.99 |
| No | 14 | 13 |  |
| PD-L1 expression |  |  |  |
| TPS<1 | 12 | 13 | 1.00 |
| TPS≥1 | 1 | 1 |  |
| Unknown | 2 | 1 |  |
| TMB (Mutation/Mb), median (Range) | 7.82 (2.79-13.4) | 8.38 (4.47-16.8) | 0.26 |
| LDH (lactate dehydrogenase, U/L), median (Range) | 199 (131-635) | 188 (142-1832) | 0.44 |

**Table S2:** Multivariate Cox regression analysis for PFS and OS in this cohort.

| Factor | Category | PFS | | OS | |
| --- | --- | --- | --- | --- | --- |
|  |  | HR with 95%CI | P value | HR with 95%CI | P value |
| Age | >61 vs. ≤61 | 1.17 (0.30-4.56) | 0.82 | 4.02 (0.61-26.4) | 0.15 |
| Sex | Male vs. Female | 0.74 (0.11-5.07) | 0.76 | 1.99 (0.03-137) | 0.75 |
| ECOG | 2 vs. 0-1 | 4.28 (0.77-23.9) | 0.10 | 0.37 (0.02-6.69) | 0.50 |
| Site | Left-side + Rectum vs. Right-side | 0.57 (0.13-2.50) | 0.46 | 0.11 (0.01-1.51) | 0.10 |
| Lung metastases | Yes vs. No | 2.00 (0.58-6.85) | 0.27 | 2.04 (0.47-8.92) | 0.34 |
| Liver metastases | Yes vs. No | **5.66 (1.58-20.2)** | **<0.01** | **7.85 (1.38-44.8)** | **0.02** |
| KRAS mutation | Yes vs. No | 1.72 (0.23-13.2) | 0.60 | 1.11 (0.05-25.7) | 0.95 |
| FLT4 mutation | Yes vs. No | 4.89 (0.50-47.4) | 0.17 | **12.52 (1.54-101)** | **0.02** |
| PIK3R1 mutation | Yes vs. No | 7.95 (0.67-94.4) | 0.10 | 3.13 (0.15-66.0) | 0.46 |
| PD-L1 | TPS >1% vs. <1% | 11.7 (0.31-441) | 0.19 | 51.0 (0.24-10671) | 0.15 |
| TMB | >8.38 vs. ≤8.38 | 1.32 (0.40-4.40) | 0.65 | 0.12 (0.01-1.10) | 0.06 |

**Table S3:** Inclusion criteria and exclusion criteria of this trial.

According to the inclusion criteria, all mCRC patients required to be treatment-naïve, unresectable and metastatic CRCs.

**Inclusion criteria**

- Patients have histologically or cytologically confirmed advanced or recurrent CRC;
- No prior systematic anti-cancer treatment and relapse or metastases was occurred more than 12 months after adjuvant chemotherapy;
- Patients have measurable disease as defined by RECIST 1.1 as determined by investigator;
- Patient with a history of radiotherapy at least 3 months before on the day of providing consent, but the measurable lesion should not be within the scope of radiotherapy;
- Patients with age of 18-75yr;
- Patients with a performance status of 0,1or 2 on the Eastern Cooperative Oncology Group.；
- Patients with Life expectancy of more than 12 weeks;
- Patients must have the ability to understand and sign the written informed consent voluntarily;
- Female of childbearing potential who are negative in a pregnancy test within 7 days before enrollment. Both male and female patients should agree to use an adequate method of contraception (total abstinence, an intrauterine device or hormone releasing system, a contraceptive implant and an oral contraceptive) starting with the first dose of study therapy through 120 days after the last dose of study therapy. Duration will be determined when the subject is assigned to treatment.

**Exclusion criteria**

- Patients with dMMR/MSI-H;
- Patients with major surgery or severe trauma within 4 weeks before the first medication;
- Patients with hypersensitivity to the components in the study protocol;；
- Patients who are ready to give birth or are pregnant;
- Patients with brain metastases who are unable to accurately describe the condition;
- Patients received immune-suppressive drugs 2 weeks before initial treatment (inhaled cortisol or other steroid hormones≤10 mg/day prednisone or equivalent pharmacophysiologic doses were excluded);
- Planned live attenuated vaccine within 4 weeks prior to or during study treatment;
- Patients have received anlotinib or anti-PD-1 monoclonal antibody therapy or other therapies that act on T-cell co-stimulation targets or checkpoints;
- Within 6 months prior to the start of study treatment, the following diseases appeared: myocardial infarction, severe/unstable angina, NYHA grade 2 or above congestive heart failure, poorly controlled arrhythmias, etc;
- Active hepatitis;
- Bone marrow, liver and kidney function did not meet the requirements of chemotherapy as follows:

- Neutrophil count<1,500/mm3;

- Platelet count <80,000/mm3;

- Total bilirubin >1.5-times the upper limit of normal;

-ALT/AST>2.5-times the upper limit of normal for patients without liver

metastases; (5.0-times the upper limit of normal for patients with

liver metastases)

- Creatinine >1.5-times the upper limit of normal;

- Patients with cancers other than advanced colorectal cancer within five years prior to the start of treatment in this study. Cervical carcinoma in situ, cured basal cell carcinoma and bladder epithelial tumor were excluded;
- History of substance abuse, drug use, alcohol dependence;
- Patients without legal capacity or limited civil capacity;
- Patients with autoimmune diseases or organ transplantation;
- Other situations that the investigator deemed inappropriate for enrollment;

**Table S4:** Comparison of immune cell infiltration in TIME with clinical response.

| **Characteristic** | **PD+SD, N = 16^1^** | **PR+CR, N = 9^1^** | **p-value^2^** |
| --- | --- | --- | --- |
| cd8_dns_cnt_tumor | 11.9 [0, 649] | 65.0 [4.07, 121] | 0.058 |
| cd8_dns_cnt_stroma | 61.6 [0, 355] | 14.0 [0.960, 170] | 0.141 |
| cd68cd163_plus_dns_cnt_tumor | 0.915 [0, 49.8] | 4.99 [0, 50.0] | 0.067 |
| cd68cd163_plus_dns_cnt_stroma | 2.37 [0, 152] | 7.79 [0, 83.0] | 0.887 |
| cd68cd163_minus_dns_cnt_tumor | 10.8 [0, 97.6] | 68.3 [0.390, 256] | 0.126 |
| cd68cd163_minus_dns_cnt_stroma | 31.3 [0, 390] | 54.0 [0.780, 680] | 0.799 |
| pd_l1cd68_plus_dns_cnt_tumor | 0 [0, 9.06] | 1.00 [0, 33.0] | 0.006 |
| pd_l1cd68_plus_dns_cnt_stroma | 0.0650 [0, 13.1] | 0 [0, 19.0] | 0.596 |
| cd56bright_dns_cnt_tumor | 0 [0, 305] | 0 [0, 40.0] | 0.274 |
| cd56bright_dns_cnt_stroma | 0 [0, 357] | 0 [0, 115] | 0.043 |
| cd56dim_dns_cnt_tumor | 0 [0, 1210] | 0 [0, 78.0] | 0.393 |
| cd56dim_dns_cnt_stroma | 0 [0, 696] | 0.300 [0, 79.0] | 0.138 |
| pd_1_dns_cnt_tumor | 8.10 [0, 110] | 33.0 [0.770, 201] | 0.074 |
| pd_1_dns_cnt_stroma | 21.1 [0, 368] | 10.7 [0.540, 467] | 0.755 |
| cd20_dns_cnt_tumor | 1.95 [0, 238] | 18.2 [0.440, 78.0] | 0.01 |
| cd20_dns_cnt_stroma | 8.46 [0, 271] | 25.9 [0, 351] | 0.476 |
| cd3_dns_cnt_tumor | 219 [31.2, 1970] | 285 [74.5, 1470] | 0.357 |
| cd3_dns_cnt_stroma | 511 [0, 1630] | 275 [1.91, 1640] | 0.388 |
| foxp3_dns_cnt_tumor | 23.2 [0, 117] | 41.8 [0.320, 4190] | 0.187 |
| foxp3_dns_cnt_stroma | 62.4 [2.77, 935] | 80.2 [0.0600, 529] | 0.846 |
| cd8pd_1_plus_dns_cnt_tumor | 0 [0, 7.00] | 1.71 [0, 10.2] | 0.002 |
| cd8pd_1_plus_dns_cnt_stroma | 0 [0, 5.33] | 0.0300 [0, 11.2] | 0.334 |
| tls_dns_cnt | 0 [0, 0.0800] | 0 [0, 0.910] | 0.138 |
| ^1^ Median (Range); n (%) |  |  |  |
| ^2^ Wilcoxon rank sum test; Wilcoxon rank sum exact test |  |  |  |

**Table S5:** Antibodies used in the study

|  | Target | Source | Dilution | Opal fluorophores | Color |
| --- | --- | --- | --- | --- | --- |
| Panel 1 | CD163 | Abcam, ab182422 | 1:500 | Opal 620 | Red |
|  | CD8 | Abcam, ab178089 | 1:200 | Opal 690 | Magenta |
|  | CD68 | Abcam, ab213363 | 1:1000 | Opal 480 | Cyan |
|  | PD-1 | CST, D4W2J, 86163S | 1:200 | Opal 520 | Green |
|  | PD-L1 | CST, E1L3N, 13684S | 1:400 | Opal 570 | Yellow |
| Panel 2 | CD20 | DAKO, L26, IR604 | 1:1 | Opal 620 | Green |
|  | CD3 | DAKO, A0452 | 1:1 | Opal 690 | Magenta |
|  | CD56 | Abcam, ab75813 | 1:1000 | Opal 480 | Cyan |
|  | CD4 | Abcam, ab133616 | 1:100 | Opal 520 | Red |
|  | FOXP3 | Abcam, ab20034 | 1:100 | Opal 570 | Yellow |
| Detection in common | pan-CK | Abcam, ab7753 | 1:100 | Opal 780 | White |

**Method S1:** NGS detection for molecular alteration profile of each enrolled mCRC patients

**Tissue processing and genomic DNA extraction**

Formalin-fixed paraffin-embedded (FFPE) tissue sections were evaluated for tumor cell content using hematoxylin and eosin (H&E) staining. Only samples with a tumor content of ≥20% were eligible for subsequent analyses. FFPE tissue sections were placed in a 1.5 microcentrifuge tube and deparaffinized with mineral oil. Samples were incubated with lysis buﬀer and proteinase K at 56 ° C overnight until the tissue was completely digested. The lysate was subsequently incubated at 80 °C for 4 hours to reverse formaldehyde crosslinks. Genomic DNA was isolated from tissue samples using the ReliaPrep™ FFPE gDNA Miniprep System (Promega) and quantified using the Qubit™ dsDNA HS Assay Kit (Thermo Fisher Scientific) following the manufacturer’s instructions.

**Library preparation and targeted capture**

DNA extracts (30-200 ng) were sheared to 250 bp fragments using an S220 focused-ultrasonicator (Covaris). Libraries were prepared using the KAPA Hyper Prep Kit (KAPA Biosystems) following the manufacturer’s protocol. The concentration and size distribution of each library were determined using a Qubit 3.0 fluorometer (Thermo Fisher Scientific) and a LabChip GX Touch HT Analyzer (PerkinElmer) respectively.

For targeted capture, indexed libraries were subjected to probe-based hybridization with a customized NGS panel targeting 733 cancer-related genes, where the probe baits were individually synthesized 5′ biotinylated 120 bp DNA oligonucleotides (IDT). Repetitive elements were filtered out from intronic baits according to the annotation by UCSC Genome RepeatMasker [1]. The xGen® Hybridization and Wash Kit (IDT) was employed for hybridization enrichment. Briefly, 500 ng indexed DNA libraries were pooled to obtain a total amount of 2 μg of DNA. The pooled DNA sample was then mixed with human cot DNA and xGen Universal Blockers-TS Mix and dried down in a SpeedVac system. The Hybridization Master Mix was added to the samples and incubated in a thermal cycler at 95℃ for 10 min, before being mixed and incubated with 4 μl of probes at 65℃ overnight. The target regions were captured following the manufacturer’s instructions. The concentration and fragment size distribution of the final library were determined using a Qubit 3.0 fluorometer (Thermo Fisher Scientific) and a LabChip GX Touch HT Analyzer (PerkinElmer) respectively.

**DNA sequencing, data processing, and variant calling (for tissue-based testing)**

The captured libraries were loaded onto a NovaSeq 6000 platform (Illumina) for 100 bp paired-end sequencing with a mean sequencing depth of 1000.

Raw data of paired samples (an FFPE sample and its normal tissue control) were mapped to the reference human genome hg19 using the Burrows-Wheeler Aligner (v0.7.12) [2]. PCR duplicate reads were removed and sequence metrics were collected using Picard (v1.130) and SAMtools (v1.1.19), respectively. Variant calling was performed only in the targeted regions. Somatic single nucleotide variants (SNVs) were detected using an in-house developed R package to execute a variant detection model based on binomial test. Local realignment was performed to detect indels. Variants were then filtered by their unique supporting read depth, strand bias, base quality as previously described [3]. All variants were then filtered using an automated false positive filtering pipeline to ensure sensitivity and specificity at an allele frequency (AF) of ≥ 1%. Single-nucleotide polymorphism (SNPs) and indels were annotated by ANNOVAR against the following databases: dbSNP (v138), 1000Genome and ESP6500 (population frequency > 0.015). Only missense, stopgain, frameshift and non-frameshift indel mutations were kept. Copy number variations (CNVs) and gene rearrangements were detected as described previously [3].

Tumor mutational burden was defined as the number of non-synonymous somatic SNVs and indels in examined coding regions, with driver mutations excluded. All SNVs and indels in the coding region of targeted genes, including missense, silent, stop gain, stop loss, in-frame and frameshift mutations, were considered.

MATH score was calculated based on the MAF files using ‘maftools’ packages of R.

The “maftools” package was performed to examine the genomic landscape. Copy number variation analysis was performed using an in-house developed pipeline. A fold change threshold of 1.6 and 0.6 in DNA copy number was set as the cut-off for amplification and deletion, respectively. The key pathway-related genes were visualized, including HGF signaling, EGFR/RAS/BRAF signaling, CDK signaling, AKT/mTOR/PI3K signaling, FGFR signaling, p53 signaling, epigenetics/chromatin remodelin, DNA damage and repair /Telomere stability, NOTCH signaling. other clinical trial drug target and TERT promoter hot spot mutations were also showed in the genomic landscape.

**Reference:**

1. Karolchik, D. *et al*.The UCSC Table Browser data retrieval tool. *Nucleic Acids Res*, **32**(Database issue), p. D493-6(2004).

2. Li, H., Durbin, R. Fast and accurate short read alignment with Burrows-Wheeler transform. *Bioinformatics* **25**, p. 1754-60(2009).

3. Su, D. *et al*. High performance of targeted next generation sequencing on variance detection in clinical tumor specimens in comparison with current conventional methods. *J Exp Clin Cancer Res* **36**, p. 121(2017).

**Method S2:** Tumor microenvironment (TME) by multiplex immunofluorescence (mIF).

**Tumor microenvironment (TME) by multiplex immunofluorescence (mIF)**

Multiplex immunofluorescence staining was conducted using the Akoya OPAL Polaris 7-Color Automation IHC kit (NEL871001KT). FFPE tissue slides were first deparaffinized in a BOND RX system (Leica Biosystems) and then incubated sequentially with primary antibodies targeting CD163 (Abcam, ab182422, 1:500), CD68 (Abcam, ab213363, 1:1000), PD-1 (CST, D4W2J, 86163S, 1:200), PD-L1 (CST, E1L3N, 13684S, 1:400)， CD3 (Dako, A0452), CD4 (Abcam, ab133616, 1:100), CD8 (Abcam, ab178089, 1:100), CD56 (Abcam, ab75813, 1:100), CD20 (Dako, L26, IR604), FOXP3 (Abcam, ab20034, 1:100) and pan-CK ( Abcam, ab7753, 1:100) or S100 (Abcam, ab52642, 1:200) (Akoya Biosciences). This was followed by incubation with secondary antibodies and corresponding reactive Opal fluorophores. Nuclei acids were stained with DAPI. Tissue slides that were bound with primary and secondary antibodies but not fluorophores were included as negative controls to assess autofluorescence. Multiplex stained slides were scanned using a Vectra Polaris Quantitative Pathology Imaging System(Akoya Biosciences) at 20 nm wavelength intervals from 440 nm to 780 nm with a fixed exposure time and an absolute magnification of ×200. All scans for each slide were then superimposed to obtain a single image. Multilayer images were imported to inForm v.2.4.8 (Akoya Biosciences) for quantitative image analysis. Tumor parenchyma and stroma were differentiated by Pan-CK staining. The quantities of various cell populations were expressed as the number of stained cells per square millimeter and as the percentage of positively stained cells in all nucleated cells.

**Method S3:** Plasma exosomal miRNA detection

**Small RNA sequencing and bioinformatics analysis**

The isolation of circulating sEVs was performed as previous reports (PMID: 31579436). Circulating sEVs miRNAs were isolated by the miRNeasy Serum/Plasma Kit according to the manufacturer’s instructions (217184, QIAGEN, Shanghai, China). A NEBNext, Multiplex Small RNA Library Prep Set for Illumina (E7300L, New England Biolabs, Ipswich, MA, USA) was used for small RNA library construction. The libraries were sequenced using an Illumina NovaSeq 6000 analyzer. The 3’ adaptors of raw data were trimmed. Clean reads were aligned to a human genome (GRCh37), annotated using Gencode (v25) and miRBase (v21)[1, 2]. The miRNA expressions were quantified by counting the reads mapped to mature miRNAs and then normalized with the expression of miRNA located in the upper quartile of each sample. Differences in miRNA expression between PR and SD/PD groups were assessed using both the Mann-Whitney U test and edgeR, respectively[3]. DEMs were identified by determining the intersection of the results (absolute value of log fold-change greater than or equal to 1 and p-value less than or equal to 0.05). The Kyoto Encyclopedia of Genes and Genomes (KEGG) pathways enrichment of the target genes of miRNAs were examined using miRPath v.4[4].

**Reference:**

1. Griffiths-Jones, S., Saini, H.K., van Dongen, S., Enright, A,J. miRBase: tools for microRNA genomics . *Nucleic Acids Res* **36**(Database issue), D154-8 (2008).

2. Harrow, J. *et al*. GENCODE: the reference human genome annotation for The ENCODE Project. *Genome Res* **22**, 1760-74(2012).

3. Robinson, M.D., McCarthy, D.J., Smyth, G.K. edgeR: a Bioconductor package for differential expression analysis of digital gene expression data. *Bioinformatics* **26**, 139-40 (2010).

4. Tastsoglou, S. *et al*. DIANA-miRPath v4.0: expanding target-based miRNA functional analysis in cell-type and tissue contexts. *Nucleic Acids Res* **51**, W154-W9(2023).
